# Genomic insights on heterogeneous resistance to vancomycin and teicoplanin in Methicillin-resistant *Staphylococcus aureus*: A first report from South India

**DOI:** 10.1371/journal.pone.0227009

**Published:** 2019-12-30

**Authors:** Yamuna Devi Bakthavatchalam, Priyanka Babu, Elakkiya Munusamy, Hariharan Triplicane Dwarakanathan, Priscilla Rupali, Marcus Zervos, Peter John Victor, Balaji Veeraraghavan

**Affiliations:** 1 Department of Clinical Microbiology, Christian Medical College, Vellore, India; 2 Department of Orthopaedics, Christian Medical College, Vellore, India; 3 Infectious Diseases Training and Research Center (IDTRC), Christian Medical College, Vellore, India; 4 Infectious Diseases, Henry Ford Health System, Detroit, Michigan, United States of America; 5 Department of critical care unit, Christian Medical College, Vellore, India; Indiana University School of Medicine-Northwest, UNITED STATES

## Abstract

Methicillin-resistant *Staphylococcus aureus* (MRSA) infection is an important clinical concern in patients, and is often associated with significant disease burden and metastatic infections. There is an increasing evidence of heterogeneous vancomycin-intermediate *S*. *aureus* (hVISA) associated treatment failure. In this study, we aim to understand the molecular mechanism of teicoplanin resistant MRSA (TR-MRSA) and hVISA. A total of 482 MRSA isolates were investigated for these phenotypes. Of the tested isolates, 1% were identified as TR-MRSA, and 12% identified as hVISA. A highly diverse amino acid substitution was observed in *tca*RAB, *vra*SR, and *gra*SR genes in TR-MRSA and hVISA strains. Interestingly, 65% of hVISA strains had a D148Q mutation in the *gra*R gene. However, none of the markers were reliable in differentiating hVISA from TR-MRSA. Significant *pbp*2 upregulation was noted in three TR-MRSA strains, which had teicoplanin MICs of 16 or 32 μg/ml, whilst significant *pbp*4 downregulation was not noted in these strains. In our study, multiple mutations were identified in the candidate genes, suggesting a complex evolutionary pathway involved in the development of TR-MRSA and hVISA strains.

## Introduction

Methicillin-resistant *Staphylococcus aureus* (MRSA) infection is an important clinical concern in patients and is often associated with significant disease burden and metastatic infections. The relative morbidity and mortality of MRSA infections are two-fold higher than that of methicillin-susceptible *S*. *aureus* (MSSA) infections [1]. Vancomycin is often recommended as the antibiotic of the first choice. An appropriate vancomycin prescription requires a susceptible minimum inhibitory concentration (MIC) of ≤ 1.5 μg/ml to avoid treatment failure. The Clinical Laboratory Standards Institute (CLSI) guidelines suggest *S*. *aureus* strains with a vancomycin MIC of ≤ 2 μg/ml should be classified as susceptible; 4–8 μg/ml as intermediate and ≥ 8 μg/ml as resistant [2]. The European Committee on Antimicrobial Sensitivity Testing (EUCAST) guidelines, defines the vancomycin MIC breakpoint of > 2 μg/ml for non-susceptible *S*. *aureus* strains [3]. In contrast to vancomycin, widely different teicoplanin susceptibility breakpoints have been defined by CLSI (≤ 8 μg/ml) and EUCAST (≤ 2 μg/ml) guidelines [2,3]. Therefore, strains with vancomycin or teicoplanin MIC of > 2 μg/ml reflect a higher likelihood of clinical treatment failure. [3,4].

A higher vancomycin and teicoplanin MIC of ≥ 1.5 μg/ml has been linked to poor clinical outcomes in patients with MRSA bacteremia [5–7]. In recent years, heterogeneous vancomycin-intermediate *S*. *aureus* (hVISA) and vancomycin-intermediate *S*. *aureus* (VISA) have been frequently reported worldwide [8–11]. The hVISA phenotype contains a subpopulation of cells expressing high vancomycin MIC. These subpopulations are present at the approximate frequencies of 10^−4^ to 10^−6^. [12,13]. Studies have suggested that hVISA infections are associated with persistent bacteremia, treatment failure, and poor outcomes [14]. Notably, teicoplanin resistant MRSA (>8 μg/ml) has been documented with a gradual rise in vancomycin MIC (2 to 4 μg/ml) [15]. However, *van*A mediating high-level vancomycin resistance in *S*. *aureus* is rare.

CLSI-recommended MIC testing methods [16], of broth microdilution (BMD) and agar dilution (AD) method are reported to have sub-optimal sensitivity in detecting hVISA and heterogeneous resistance to teicoplanin [15]. These subpopulations grow slowly with characteristic features of pin-pointed colonies, loss of pigmentation, and change in haemolytic pattern [17]. However, an optimal method is not available for the reliable detection of hVISA in clinical isolates due to i) the multiple and complex molecular bases of hVISA, ii) there being no single specific molecular marker for detection because of the possibility of sequential accumulation of chromosomal mutations and iii) the unpredictable phenotypic expression of hVISA, which is significantly influenced by several technical parameters including variable time frame and inoculum sizes [8].

Many reports have linked cell wall thickening, reduced autolysis, and decreased surface anionic charges with hVISA/VISA subpopulations [18]. Excess of D-Ala-D-Ala targets in the cell wall serves as a molecular sink which impedes the penetration of vancomycin towards pentapeptide targets [19]. Further, the hVISA/VISA phenotypes are associated with mutations in the *vra*SR (vancomycin resistance associated sensor/regulator), *gra*SR (glycopeptide resistance–associated sensor/regulator), and *wal*KR (sensor protein kinase/regulator) genes of two component systems (TCS) [17]. Studies have also reported the upregulation of *vra*SR in hVISA/VISA, which is under the regulation of *yvqF*, a cell wall stimulon that responds to cell wall active antibiotics [20]. An amino acid substitution (H481Y/ H481N) in *rpo*B, results in an elevated surface membrane charge. This is attributed to cause cross-resistance between vancomycin and daptomycin, which are functionally cationic molecules [17].

The development of resistance to teicoplanin on therapy is occasionally described in MRSA cases [9,21]. MRSA strains resistant to teicoplanin but susceptible to vancomycin are also reported in the literature [22]. Few studies have tracked the genetic basis of teicoplanin resistance in MRSA [23–26]. The presence of a teicoplanin resistance operon (*tca*RAB) or the inactivation of *tca*A are reported to be associated with teicoplanin resistance in *S*. *aureus* [25–27].

For treating MRSA infections, vancomycin is the antibiotic of the first choice, but teicoplanin is considered in cases of osteomyelitis and septic arthritis. Teicoplanin is not approved for use in the United States, while commonly used in Europe. Observational studies and case reports documented that, in *S*. *aureus*, teicoplanin resistance emerges earlier than vancomycin resistance. [28, 29, 15]. Vancomycin susceptible revertant of VISA/VRSA can able to maintain the intermediate level of teicoplanin resistance. Acquisition of teicoplanin resistance is frequently accompanied by a small increase in vancomycin resistance [30]. An *in-vitro* study has demonstrated that overexpression of PBP2 in *S*. *aureus* increases vancomycin MIC from 1 to 2 μg/ml and teicoplanin MIC from 2 to 8 μg/ml [31]. Thus, extrapolating vancomycin MICs for teicoplanin therapy may result in therapeutic failure. In fact, patients who have failed teicoplanin therapy have been successfully treated with vancomycin [29].

The present study aims are i) to describe the phenotypic characterisation of teicoplanin resistant MRSA (TR-MRSA) and hVISA isolated from bloodstream infections, ii) to identify amino acid substitutions in the candidate genes that are associated with the development of these phenotypes, and iii) to determine the genotypes of TR-MRSA and hVISA.

## Materials and methods

### Bacterial strains

A total of 482 MRSA isolates recovered from blood culture samples during 2013–2017, were included in this study. All *S*. *aureus* strains were identified using standard microbiological methods using gram staining, catalase and coagulase tests [32]. All isolates were stored in tryptic soy broth containing 80% (v/v) glycerol at -70⁰C. The study was conducted at a 2,600 bedded tertiary care hospital, Christian Medical College, Vellore.

### Antimicrobial susceptibility testing

Methicillin resistance in *S*. *aureus* strains was detected using cefoxitin (30 μg) disc diffusion method as per the CLSI recommendation [2]. For all MRSA isolates, the MIC of vancomycin and teicoplanin was determined using the broth microdilution method (BMD) [16]. *S*. *aureus* ATCC 29213 was used as an internal quality control strain. Isolates with a teicoplanin MIC of > 2 μg/ml are referred to as TR-MRSA.

### Screening of MRSA isolates for hVISA

**Fig 1:**
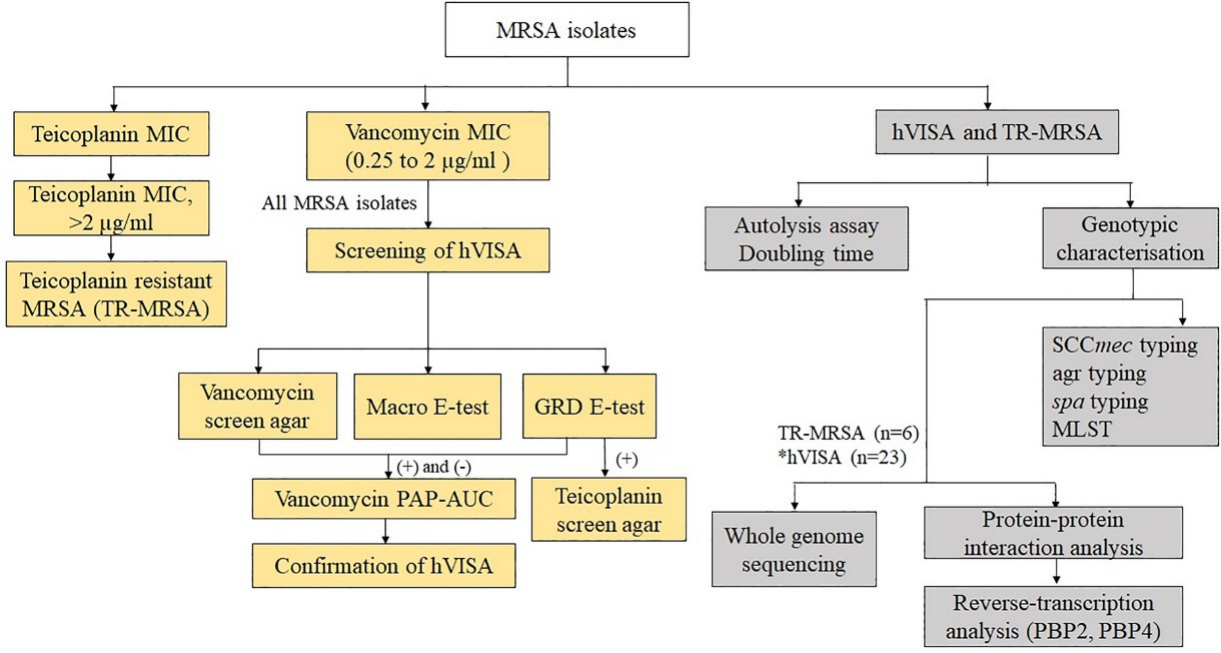
Algorithm used to test MRSA (n = 482) isolates for the presence of heterogeneous vancomycin-intermediate *S*. *aureus* (hVISA) subpopulation and heterogenous resistance to teicoplanin. hVISA—heterogenous vancomycin-intermediate *S*. *aureus*; GRD—Glycopeptide resistance detection; PAP-AUC—Population analysis profile-Area under Curve method; SCC*mec*—Staphylococcal cassette chromosome *mec; agr—*accessory gene regulator; *spa*–Staphylococcal protein A; MLST–Multilocus sequence typing; PBP- penicillin-binding protein.

The detailed flowchart representing the screening, confirmation, and molecular characterisation of hVISA and TR-MRSA is shown in Fig 1.

#### Vancomycin screen agar

All MRSA isolates were preliminarily screened for the presence of hVISA subpopulation using brain heart infusion agar (BHIA) containing 3 μg/ml (BHIV3) and 4 μg/ml (BHIV4) of vancomycin [29]. The colony forming unit (CFU) per droplet were counted as suggested by Khatib *et al*. [33].

#### Macro E-test (MET)

MET was performed as described by Satola *et al*. [34]. Bacterial suspension of colonies from an overnight culture on blood agar was prepared in Mueller-Hinton broth (MHB). Turbidity was adjusted to the McFarland standard of 2.0. A volume of 200μl of the inoculum was plated onto BHIA and swabbed evenly on the agar surface. Vancomycin (0.016 to 256 μg/ml) and teicoplanin (0.016 to 256 μg/ml) E-test strips (bioMérieux, Marcy-l'Étoile, France) were placed onto the inoculated BHIA and incubated at 37⁰C for 48 hrs. The test isolate was considered positive for hVISA, if the MIC for teicoplanin alone was ≥ 12 μg/ml or if the MIC for teicoplanin and vancomycin was ≥8 μg/ml.

#### Glycopeptide resistance detection (GRD) E-test

GRD E-test (bioMérieux, Marcy-l'Étoile, France) was performed, according to the manufacturer instructions. This E-test strip contains a double-sided pre-defined gradient of vancomycin (0.5–32 μg/ml) and teicoplanin (0.5–32 μg/ml). A suspension of bacterial colonies from an overnight culture on blood agar was prepared in MHB, and the turbidity was adjusted to the McFarland standard of 0.5. The inoculum was swabbed onto a Mueller-Hinton agar (MHA) plate containing 5% blood and incubated at 37 ± 2⁰C for 24–48 hrs. The plates were examined at 24 hrs and 48 hrs. The test isolate was considered positive for hVISA, if the MIC of vancomycin or teicoplanin was ≥ 8 μg/ml.

All GRD positive MRSA isolates were screened for heterogeneous resistance to teicoplanin. A 10 μl of 0.5 Mc Farland adjusted bacterial inoculum was plated on to the BHIA plates containing teicoplanin (4–16 μg/ml) and incubated for 48 hrs. The MIC for vancomycin and teicoplanin was retested for colonies which grew on the plates containing 4–16 μg/ml of teicoplanin.

#### Population analysis profile-area under curve (PAP-AUC) analysis

Modified PAP-AUC analysis was performed as described by Wootton *et al*. [35]. The bacterial strains were incubated in tryptic soy broth (TSB) at 37⁰C for 18–24 hrs. After incubation, the inoculum was diluted 1 in 1000 in saline, 10^−3^ to 10^−6^. The bacterial suspension was plated onto freshly prepared BHIA plates containing 0.5 to 8 μg/ml of vancomycin. Colonies were counted after 48 hrs incubation at 35°C ± 2. For calculation of AUC, viable counts were plotted against increasing concentration of vancomycin using the GraphPad Prism^TM^ (V.7.0) software package. All the PAP-AUC experiments were performed in duplicate. For the vancomycin PAP analysis, the AUC ratio was calculated by dividing the AUC of the test strain by the AUC of the reference MU3 (hVISA) strain. The PAP-AUC ratio was interpreted as follows, <0.9 as vancomycin-susceptible *S*. *aureus* (VSSA), ≥ 0.9 as hVISA phenotype, >1.3 as vancomycin-intermediate *S*. *aureus* (VISA). For all the PAP-AUC experiments, the hVISA (MU3, ATCC 700698), VISA (MU50, ATCC 700699), and *S*. *aureus* ATCC 29213 (VSSA) were used as the reference, comparator, and negative control strains, respectively.

### Autolysis assay

Lysis of MRSA strains to Triton X-100 has been used to evaluate the autolytic activity as described by Rodriguez *et al*. [36]. Cells were grown to a log-phase to an OD_600_ of about 0.3. The cultures were then rapidly chilled and centrifuged at 14,000 rpm for 10 minutes at 4⁰C. The cell pellet was washed twice with ice-cold water and resuspended in 50 mM Tris/HCl (pH 7.5) containing 0.1% Triton X-100, to achieve an OD_600_ of 1.0. This suspension was incubated at 37⁰C for 4 hrs, and the absorbance was measured every 30 minutes using spectrophotometry. The normalisation of the results was carried out to OD600 at time zero (OD0) using the following formula; percent lysis at a time (t) = (OD_0_-OD_t_)/ OD_0_ X 100. For all experiments, hVISA (MU3, ATCC 700698), VISA (MU50, ATCC 700699), and *S*. *aureus* ATCC 29213 (VSSA) were used as the control strains. Assay results obtained by three independent experiments were expressed as mean ± standard deviation (SD).

### Doubling time

The growth rate of TR-MRSA and hVISA were measured as described by Chen *et al*. [37]. For every 30 minutes, a change in OD was monitored for 8hrs. Doubling time of the strain was calculated using the formula; (t_2_-t_1_ x log_2_)/(log OD_600_ at t_2_- log OD_600_ at t_1_); t1 (first sampling time); t2 (second sampling time). hVISA (MU3, ATCC 700698), VISA (MU50, ATCC 700699), and *S*. *aureus* ATCC 29213 (VSSA) were used as control strains. Doubling time of the strains were obtained by three independent experiments and were expressed as mean ± SD.

### Extraction of nucleic acids

#### Genomic DNA extraction

For DNA extraction, 5–10 morphologically similar colonies from overnight blood agar plates were removed and suspended in 200μl of 1X TE containing 40mg/ml of lysozyme which was incubated at 37⁰C for an hour. The genomic DNA was extracted using the QIAamp DNA Mini Kit according to the manufacturer’s instruction. The extracted DNA was eluted in a total volume of 100 μl and stored at -70⁰C.

### Accessory gene regulator (*agr*) typing

Multiplex PCR was performed for the detection of *agr* types as described by Goudarzi *et al* [38].

### Staphylococcal cassette chromosome (SCC)*mec* typing

SCC *mec* typing was performed for all the isolates using multiplex PCR assay [39]. SCC *mec* IV subtypes were identified by performing a multiplex PCR assay [40]. Control strains of SCC*mec* types I, II, III, IV and V were procured from the Biodefense and Emerging Infections (BEI) Research Resources Repository.

### *S*. *aureus* protein A (*spa*) typing

For all the hVISA isolates, *spa* typing was performed as described by Harmsen *et al*. [41]. Amplified hypervariable region of the *spa* gene was sequenced by using applied biosystem ABI 3130 genetic analyzer. Analysis of nucleotide sequences and the assignment of *spa* type was determined using the *spa* type database Ridom SpaServer (www.spaserver.ridom.de).

### Multi-locus sequence typing (MLST)

MLST was performed on all TR-MRSA and hVISA isolates as described by Enright *et al*. [42]. The allelic profile and sequence type (ST) were defined using the MLST database (https://pubmlst.org/). MLST clonal complexes were identified using PHYLOViZ, which is restricted to the single locus variant (SLV) and the double locus variant (DLV) of the primary founder in each group [43]. MRSA lineages were indicated using the nomenclature MLST-SCC *mec* type.

### Genome sequencing, assembly, and annotation

Whole-genome sequencing analysis was performed on all TR-MRSA (*n* = 6) and randomly selected hVISA (*n* = 23) isolates using the Ion Torrent PGM platform. DNA sample concentrations were determined using the Qubit system (Invitrogen). Ion Xpress Plus fragment library kit (Thermo Fisher Scientific, Waltham, MA, USA) was used for the fragmentation of 200–500 ng of genomic DNA, to prepare 400-bp reads according to the manufacturer’s instruction. DNA libraries were purified using AMPure XP beads (Beckman Coulter, California, USA). The libraries were then enriched with Ion PGM Hi-Q OT2 kit (Life Technologies, Inc.). Subsequently, genome sequencing was performed using the Ion Torrent Personal Genome Machine platform (Life Technologies, Inc.). The sequencing reads were *de novo* assembled using Assembler SPAdes v5.0.0.0 inbuilt in Torrent suite server version 5.0.3.

*De novo* assembled sequence was further annotated by submitting the sequence to PATRIC, the bacterial bioinformatics database and analysis resource [44] and Rapid Annotation using Subsystem Technology (RAST) pipeline [45–47] and National Center for Biotechnology Information (NCBI) Prokaryotic Genome Automatic Annotation Pipeline (PGAP).

### Mutation analysis

Candidate genes in TR-MRSA and hVISA genomes were analysed for amino acid substitutions. These candidate genes include teicoplanin resistant operon (*tca*RAB), *vra*SR, *vra*T, *gra*SR, *wal*KR, and *lyt*SR (regulates the electrical potential of cell membrane); virulence regulating genes *clp*P (stress tolerance and virulence regulation), *pho*R and *sae*S (virulence regulator), *mprF* (multiple peptide resistance factor) and a *msrR* (methionine sulfoxide reductase) gene which is involved in the production of wall-teichoic acid (WTA). In addition, all the genomes were screened for the mutations in penicillin-binding protein (PBP) encoding genes (*pbp*2, *pbp4*) and a *rpo*B gene encoding for β subunit of bacterial RNA polymerase. Hereinafter, all amino acid substitutions are referred as mutations.

These candidate genes were analysed for mutations using ST/clonal complex(CC)- specific VSSA reference genome, DAR4145 (ST772, accession no. CP010526); GR1 (ST672, accession no. AJLX00000000); HO50960412 (ST22, accession no. HE681097); TW20 (ST239, accession no. FN433596); USA400-0051 (ST1, accession no. CP019574); CN1 (ST72, accession no. CP003979); MRSA252 (ST30, accession no. BX571856); N315 (ST5, accession no. BA000018); MCRF184 (ST45, accession no. CP014791).

TW20 was used as the reference genome for mutation analysis of ST368 (SLV of ST239); HO50960412 as a reference genome for ST2371 (SLV of ST22); MRSA252 (as a reference genome for ST1482 (SLV of ST30); N315 as a reference for ST6 (DLV of ST5); MCRF184 as a reference sequence for ST1290 (SLV of ST45).

### SIFT (sorting intolerant from tolerant)

SIFT (https://sift.bii.a-star.edu.sg/www/SIFT_seq_submit2.html) is a sequence-based homology approach which predicts the effect of amino acid substitution on protein function. This algorithm is based on PSI-BLAST [48]. SIFT calculates the probabilities for all possible twenty amino acids at each position of the query sequences [49]. These probabilities are verified in a scaled probability matrix. Based on this, SIFT calculates the SIFT score or tolerable index (TI) score for every position in the submitted sequences. The SIFT score ranges from 0 to 1. The amino acid substitution is considered to be deleterious, if the score is ≤ 0.05 and tolerated, if the score is > 0.05.

### Protein-Protein Interaction analysis

The protein-protein interaction analysis was carried out using Cytoscape 3.0 [50]. The basic plot of the interaction map was obtained from the STRING database [51]. The protein name was given as the input, and *S*. *aureus* was selected as the strain, and the (.tsv) file of the interaction was uploaded to the Cytoscape analysis.

### Quantitative real-time reverse transcription PCR (qRT-PCR)

The Quantitative reverse transcriptase PCR (RT-PCR) was used to determine the mRNA levels. Expression of PBP2 and PBP4 were studied using the primers and probes as described earlier [52]. Complementary DNA (cDNA) was synthesised using 1 μg of total RNA using the QuantiTect Reverse Transcription Kit (Qiagen, France). Quantitative real-time PCR was carried out using ABI fast real-time PCR system and quantiTect Probe PCR Master Mix (Qiagen, France). The concentration of each primer (1 μM) and probe (0.5μM) were used for this qRT-PCR. The difference in expressions was analysed by relative quantification with comparative ΔΔCT method using vancomycin susceptible *S*. *aureus* ATCC 29213 as the comparator [53]. Each reaction mixture contains 2μl cDNA in a reaction volume of 20μl with a final concentration of 50 ng. Transcription of PBP was considered upregulated or downregulated when mRNA was expressed at a level of 4-fold higher or lower than that of *S*. *aureus* ATCC 29213. 16s rRNA was used as an internal control. *S*. *aureus* ATCC 700698(MU3, hVISA) and *S*. *aureus* ATCC 700699 (MU50, VISA) were used as the comparator control. Based on their 16s rRNA, all mRNA levels were normalised [54]. Normalised *pbp*2 and *pbp*4 fold changes were converted to logarithmic values. All qRT-PCR experiments were performed independently three times.

According to the threshold cycle (CT) values, gene expression was compared and converted to fold change using ΔΔCT. The change in the transcript level was calculated using the formula,

Normalisation to endogenous control: Ct target gene—Ct endogenous control = ΔCt

Normalisation to the reference sample: ΔCt sample - ΔCt reference = ΔΔCt

Use the formula: Fold change = 2^-ΔΔCt^

### Ethical clearance

The study was approved by the Institutional Review Board of Christian Medical College, Vellore (IRB Min. no 8692 dated 26.02.2014). All the data or samples were fully anonymised before accessing them for further processing. This study only utilised isolates from positive blood cultures, so it doesn’t require informed written consent from the patient.

### Statistical analysis

All statistical analysis was performed using SPSS statistical software (V21.0; SPSS, Inc., Chicago, IL). Sensitivity and specificity were evaluated for BHIV3, BHIV4, MET, and GRD E-test using PAP-AUC as the reference method. Sensitivity and specificity denoted the fraction of true positives and true negatives identified by each method, respectively. Association between the categorical variables were determined using the chi-square (χ^2^) test or Fisher’s exact test. Two-tailed student t-test was used to evaluate the statistical significance of data that were expressed as mean ± standard deviation. Transcription changes of PBP2 and PBP4 in mean logarithmic values were compared by one-way analysis of variance (ANOVA) with multiple comparison using Bonferroni corrections. A p-value of < 0.05 was considered statistically significant.

## Results

All tested MRSA isolates demonstrated vancomycin MIC of ≤ 2 μg/ml. Of these, only six had teicoplanin MIC of > 2 μg/ml. The distribution of vancomycin and teicoplanin MIC is shown in S1 Fig. The MIC_50/90_ of vancomycin and teicoplanin were 0.5/1 and 1/2 μg/ml, respectively.

### Characterisation of hVISA and TR-MRSA

**Fig 2:**
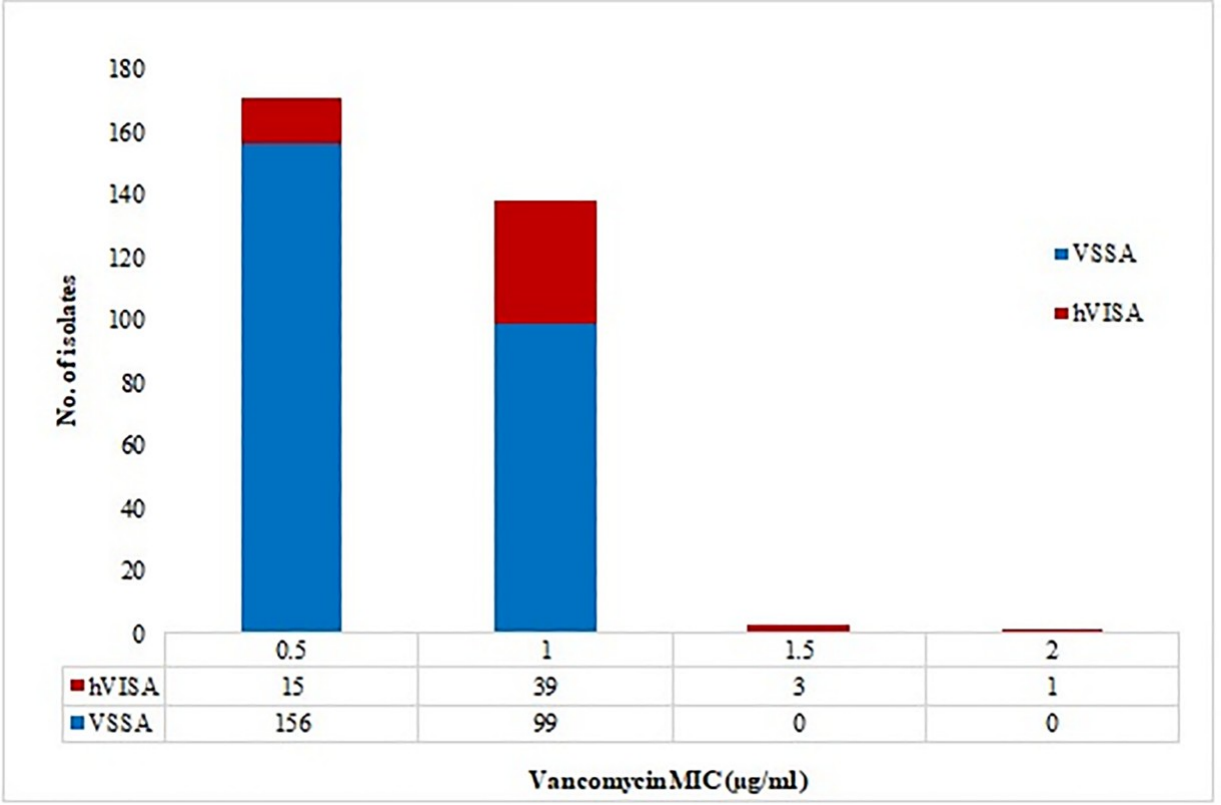
Distribution of hVISA subpopulation among vancomycin MIC of MRSA isolates recovered from bloodstream infection.

All MRSA isolates (n = 482), were screened for the presence of hVISA subpopulation. Of these, 54% (n = 260), and 26% (n = 127) showed growth on BHIV3 and BHIV4, respectively. In MET, 18% (n = 86) of isolates were found to be positive, and 16% (n = 78) of isolates were considered positive in GRD E-test. Using the vancomycin PAP-AUC method, 13% (n = 64) of the isolates were confirmed as hVISA. The proportion of PAP-AUC confirmed hVISA strains distributed among vancomycin MIC is shown in Fig 2. Compared to an MIC of 0.5 μg/ml, a higher proportion of hVISA was identified among MRSA strains which had a vancomycin MIC of 1 μg/ml (28% vs. 9%, p < 0.05). The sensitivity and specificity of the screening methods were as follows, BHIV3 (100% and 53%); BHIV4 (100% and 84%); macro E-test (100% and 94%); and GRD E-test (100% and 96%).

All GRD positive MRSA isolates were screened for heterogeneous resistance to teicoplanin. Only, six isolates which had a teicoplanin MIC of > 2 μg/ml (S1 Fig) showed growth on the teicoplanin screening agar. Retesting of these isolates showed a one-fold increase in vancomycin MIC (1 to 3 μg/ml) and a two-fold increase in teicoplanin MIC (8 to16 μg/ml for VB9352 and VB23686; 16 to 32 μg/ml for VB31683) (Table 1). Based on the teicoplanin susceptibility, these hVISA strains were classified as, i) TR-MRSA (n = 6) with teicoplanin MIC of 4–32 μg/ml; and ii) hVISA strains (n = 58), with teicoplanin MIC of 1 to 2 μg/ml. The PAP-AUC ratio of these hVISA strains is shown in S1 Table.

**Table 1:** Phenotypic and molecular characterisation of teicoplanin resistant Methicillin resistant *S*. *aureus* (TR-MRSA).

Strain ID	MIC (μg/ml)		Rif	PAP-AUC ratio	ST/SCC*mec* type	*agr* types	Mutations observed in the candidate genes or two-component system (TCS)						
	Van	Tp					*tca*A	*tca*B	*gra*S	*gra*R	*rpo*B	*lyt*S	*pho*R
VB9352	3	16	R	0.98 (hVISA)	ST772-V	I	**D230E**	**Y6R**	T224I	-	H481Y	**P315R A318Q A319L I320S** **V321M**	-
VB23686	3	16	S	1.04 (hVISA)	ST772-V	I	**D230E**	**Y6R**	T224I	-	-	-	-
VB26276	2	4	S	0.98 (hVISA)	ST772-V	II	**D230E**	**Y6R**	T224I	-	-	-	-
VB12268	3	4	S	1.02 (hVISA)	ST672-IVa	II	Y237H	H6Y	-	-	-	-	-
VB169	1.5	4	S	0.97 (hVISA)	ST672-V	II	Y237H	H6Y	-	-	-	-	-
VB31683	3	32	S	0.92 (hVISA)	ST22-IVc	I	**F290S**	-	-	D148Q	-	-	**V186I** **L144I** **V535M**

Novel mutations are in bold face; previously reported mutations are in unbold text; Van–Vancomycin; Tp–Teicoplanin; Rif–Rifampicin; PAP–population analysis profile; ST–Sequence type; SCC–staphylococcal cassette chromosome.

### Genomic characterisation of TR-MRSA and hVISA

#### Mutation analysis in TR-MRSA

All six TR-MRSA isolates were negative for both *van*A and *van*B genes. Whole-genome sequence analysis revealed several distinct mutations shown in Table 1 and no change in the sequences was seen in *tca*R, *vra*S/R, *vra*T, *wal*K/R, *lyt*R, *clp*P, *sae*S, *msr*R and *mpr*F genes. Novel mutations were identified in *tca*A (D230E, F290S) and *tca*B (Y6R). One isolate (VB9352) had H481Y substitution in *rpo*B gene, which resulted in a rifampicin MIC of > 32 μg/ml. An amino acid substitution, Y6R in the *tca*B gene, was found to be deleterious with a SIFT score of 0.001. In *lyt*S, five distinct deleterious novel substitutions were identified with the following SIFT scores; P315R (0.01), A318Q (0.001), A319L (0.001), I320S (0.001), and V321M (0.03). Similarly, a novel substitution, V535M (0.03) identified in the *pho*R gene was found to be deleterious. However, a significant association was not seen between high teicoplanin MIC and the accumulation of these mutations.

According to the identified mutations in *tca*AB, *gra*SR, *rpo*B, *lyt*S, and *pho*R genes, four different patterns were found (Table 1). TR-MRSA belonged to three different sequence types (STs), ST772, ST672 and ST22. Among these STs, mutations were frequently observed in *tca*A and *tca*B followed by *gra*S. In VB31683 (ST22), a combination of mutations in *tca*A (F290S), *gra*R (D148Q) and *pho*R (V186I, L144I, V535M) may have resulted in a teicoplanin MIC of 32 μg/ml.

#### Genotype and mutation analysis in hVISA

**Fig 3:**
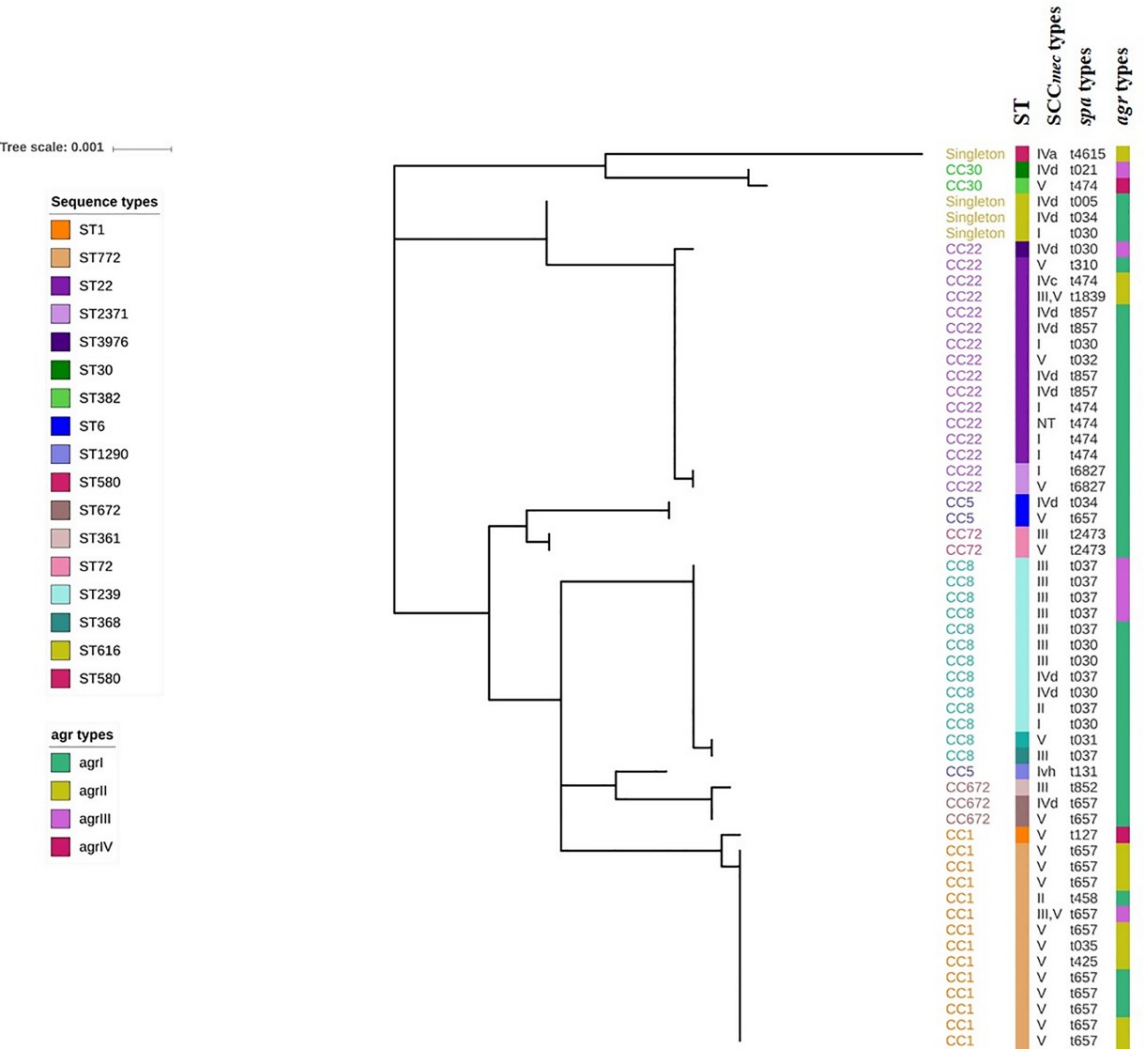
A phylogenetic tree was constructed based on the seven housekeeping MLST genes of *S*. *aureus* using MEGA V.7.0. hVISA strains belonged to different clonal complexes, sequence types and *agr* types were colour coded.

**hVISA genotype.** The genotype of hVISA was determined using SCC*mec* typing, *spa* typing, and MLST (Fig 3). Seven distinct clonal complexes (CC1, CC5, CC8, CC22, CC30, CC72, CC672) and two singletons (ST616, ST580) were identified. The most representative were CC1 (25%), CC22 (28%) and CC8 (23%). A further sixteen different STs (ST1, ST6, ST22, ST30, ST72, ST239, ST361, ST368, ST382, ST580, ST616, ST672, ST772, ST1290, ST2371, and ST3976) were identified (Fig 3). The most predominant were ST772 (23%) and ST22 (23%) followed by ST239 (19%). The most common SCC*mec* type was V (35%) followed by SCC*mec* IV (26%) and III (18%). However, *spa* types were highly diverse among these hVISA isolates. The majority of hVISA strains were detected with *agr* I (57%) followed by *agr* II (26%) and *agr* III (17%).

#### Mutations identified in hVISA

Whole-genome analysis of hVISA (n = 23) revealed distinct amino acid substitutions in eleven candidate genes (Table 2). However, none of the tested isolates showed mutations in *tca*R, *vra*T, *wal*K/R, *lyt*S, *clp*P or *msr*R genes. Several novel mutations were identified in *tca*A (D230E, F290S), *tca*B (L173M), *vra*R (T24K), *gra*S (T224K), *rpo*B (N474S, L466S, S486L), *lyt*R (G122D, T118N, N125S), *sae*S (I340M, L203V), *pho*R (V535M) and *mpr*F (T635I, E709D). Of these, the substitutions N474S in *rpo*B and V535M in *pho*R were found to be deleterious with a SIFT score of 0.001.

**Table 2:** PAP-AUC analysis and amino acid substitutions observed in the candidate genes of hVISA isolated from bloodstream infection.

Strain ID	Van MIC (μg/ml)/ PAP-AUC ratio	Rif	Mutations observed in the candidate genes or two-component system (TCS)										
			*tca*A	*tca*B	*vra*S	*vra*R	*gra*S	*gra*R	*rpo*B	*lyt*R	*sae*S	*pho*R	*mpr*F
VB9939	1/0.91 (hVISA)	S	**D230E** Y237H	-	-	-	T224I	-	-	-	-	-	-
VB16578	1/0.96 (hVISA)	S	**D230E**	-	-	-	T224I	-	-	-	-	**V535M**	-
VB46389	1/1.03 (hVISA)	S	**D230E**	-	-	**T24K**	T224I	D148Q	-	-	-	**V535M**	-
VB7336	1/0.99 (hVISA)	S	**D230E**	-	-	**T24K**	-	D148Q	-	-	-	**V535M**	-
VB13872	1/0.95 (hVISA)	S	**D230E**	-	-	-	-	D148Q	-	-	-	**V535M**	-
VB14468	1/0.97 (hVISA)	S	**D230E**	-	-	-	-	-	-	-	-	**V535M**	-
VB14915	1/1.03 (hVISA)	S	**D230E**	-	-	-	-	-	-	-	-	**V535M**	-
VB103	1/1.0 (hVISA)	S	**D230E**	-	**-**	-	T224I	D148Q	-	-	-	-	-
VB1919	1/0.92 (hVISA)	S	**F290S**	-	-	-	-	D148Q	-	-	-	-	-
VB44094	1.5/1.23 (hVISA)	S	**F290S**	-	-	-	-	D148Q	-	-	-	**V535M**	**T635I** **E709D**
VB25679	2/1.27 (hVISA)	S	**F290S**	-	-	-	-	D148Q	-	-	**I340M**	-	-
VB9882	1.5/1.03 (hVISA)	R	**F290S**	-	-	-	-	D148Q	**N474S**	-	**I340M**	**V535M**	**T635I**
VB20017	1.5/0.96 (hVISA)	S	**F290S**	*-*	*-*	-	T224I	D148Q	-	-	**I340M**	-	**T635I**
VB1490	1/1.01 (hVISA)	S	*-*	*-*	*-*	-	L26F I59L T224I	D148Q	-	**G122D**	-	-	-
VB9190	1/1.1 (hVISA)	R	*-*	*-*	V15G	-	L26F I59L R232K	D148Q	H481N L466S	**G122D**	**L203V**	-	-
VB14511	1/0.92 (hVISA)	S	*-*	*-*	V15G	-	L26F, I59L	D148Q	-	**G122D**	**L203V**	-	-
VB43011	1/1.28 (hVISA)	R	*-*	*-*	*-*	-	T224I	-	**S486L**	-	-	-	-
VB44746	1/1.26 (hVISA)	S	*-*	*-*	*-*	-	L26F T224I	-	-	-	-	-	-
VB4283	1/1.25 (hVISA)	S	*-*	**L173M**	*-*	-	T224I	D148Q	-	-	-	-	-
VB7185	1/0.9 (hVISA)	S	*-*	*-*	*-*	-	L26F I59L T224I	D148Q	-	-	**L203V**	-	-
VB43964	1/1.02 (hVISA)	S	*-*	*-*	*-*	-	T224I	**D147E** D148Q	-	-	-	-	-
VB3985	1/1.14 (hVISA)	S	L218P G312D	K396R	**H6Y** **K396R**	-	L26F, **T224K**	-	-	**T118N** **N125S**	-	-	-
VB35316	1/0.96 (hVISA)	S	*-*	*-*	*-*	-	T224I	-	-	**E31D**	-	-	-

Novel mutations are in bold face; previously reported mutations are in unbold text; Van–Vancomycin Rif–Rifampicin; PAP–population analysis profile

Based on the identified mutations in hVISA, 26 different patterns were identified (S2 Table). Among hVISA, D148Q (65%) in *gra*R was identified as the predominant mutation followed by T224I (52%) substitution in *gra*S, and D230E (35%) in *tca*A. A strong link was seen between hVISA phenotype and the mutations identified in *tca*A (D230E), *gra*S (T224I) and *gra*R (D148Q) (p <0.05). Of these mutations, T224I and D148Q were identified in various STs (S2 Table) and D230E substitution was most commonly seen among the hVISA strains which belonged to ST772.

#### Difference in the mutation between TR-MRSA and hVISA

A large number of mutations were identified in the candidate genes of TR-MRSA and hVISA (Tables 1 and 2). Many non-overlapping mutations were identified in both TR-MRSA and hVISA (S3 Table). However, mutations in *tca*A (D230E, Y237H, F260S), *gra*S (T224I) and *gra*R (D148Q) were shared by both TR-MRSA and hVISA.

### Triton X-100 induced autolysis

**Fig 4:**
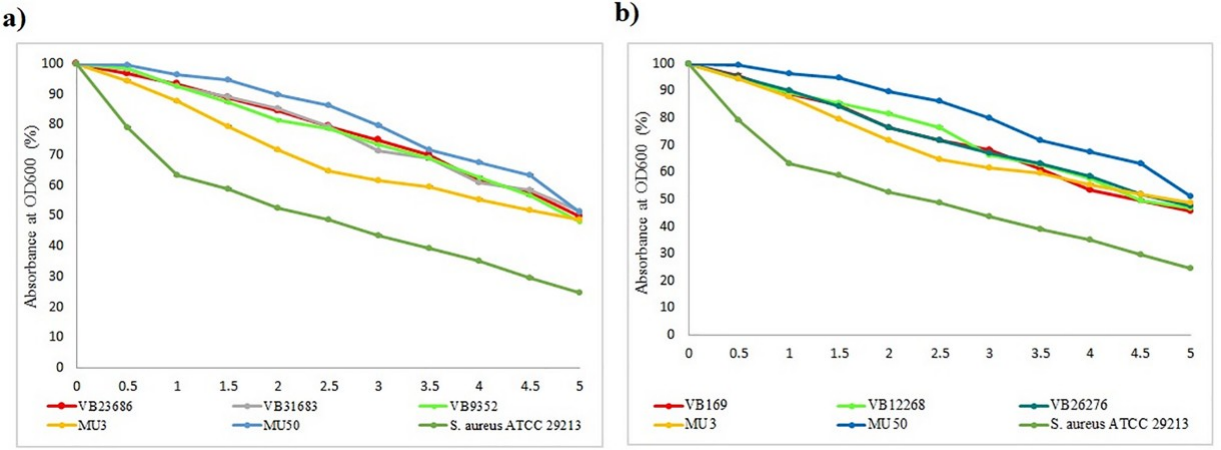
Triton X-100 induced autolysis in teicoplanin resistant MRSA (TR-MRSA). Lysis of cells was measured as the percentage of absorbance measured at OD600nm. The data are represented as mean ± mean standard deviation (SD). a) Triton X-100 induced autolysis in TR-MRSA with MICs of 16 and 32 μg/ml; b) Triton X-100 induced autolysis in TR-MRSA with MIC of 4 μg/ml. The autolysis rate of all TR-MRSA strains was significantly higher than *S*. *aureus* ATCC 29213 (p <0.05). VB9352 and VB31683 showed decreased lytic activity than MU3 (p <0.01).

The autolysis rate of TR-MRSA to triton X-100 is shown in Fig 4. TR-MRSA strains showed significantly decreased autolytic activity, as compared to *S*. *aureus* ATCC 29213 (p <0.05) (S4 Table). However, only two TR-MRSA strains (VB9352, VB31683) showed decreased autolytic activity compared to MU3 (p <0.01). The autolysis rate observed in three TR-MRSA strains (VB23686, VB31683, VB9352) were almost comparable to that of the MU50 strain. Triton X-100 induced autolytic rate of hVISA strains are shown in the S5 Table. Although hVISA strains showed decreased autolytic rates, this was not significant as compared to *S*. *aureus* ATCC 29213.

### Doubling time

The doubling times of TR-MRSA, ranged from 33.7 to 37.7 minutes. TR-MRSA strains (VB23686, VB31683, VB9352) had a significantly longer doubling times (p < 0.05) than *S*. *aureus* ATCC 29213 (S4 Table). The doubling time of these strains was almost comparable to MU3 but was shorter than for the MU50 strain. Among hVISA, the doubling time ranged from 32.4 to 36.8 minutes. Nineteen hVISA strains had a significantly longer doubling time than *S*. *aureus* ATCC 29213 (p < 0.05) (S5 Table). All these hVISA strains had a doubling time, comparable to that of the MU3 strain.

### Protein network analysis of TCS involved in cell wall biosynthesis

**Fig 5:**
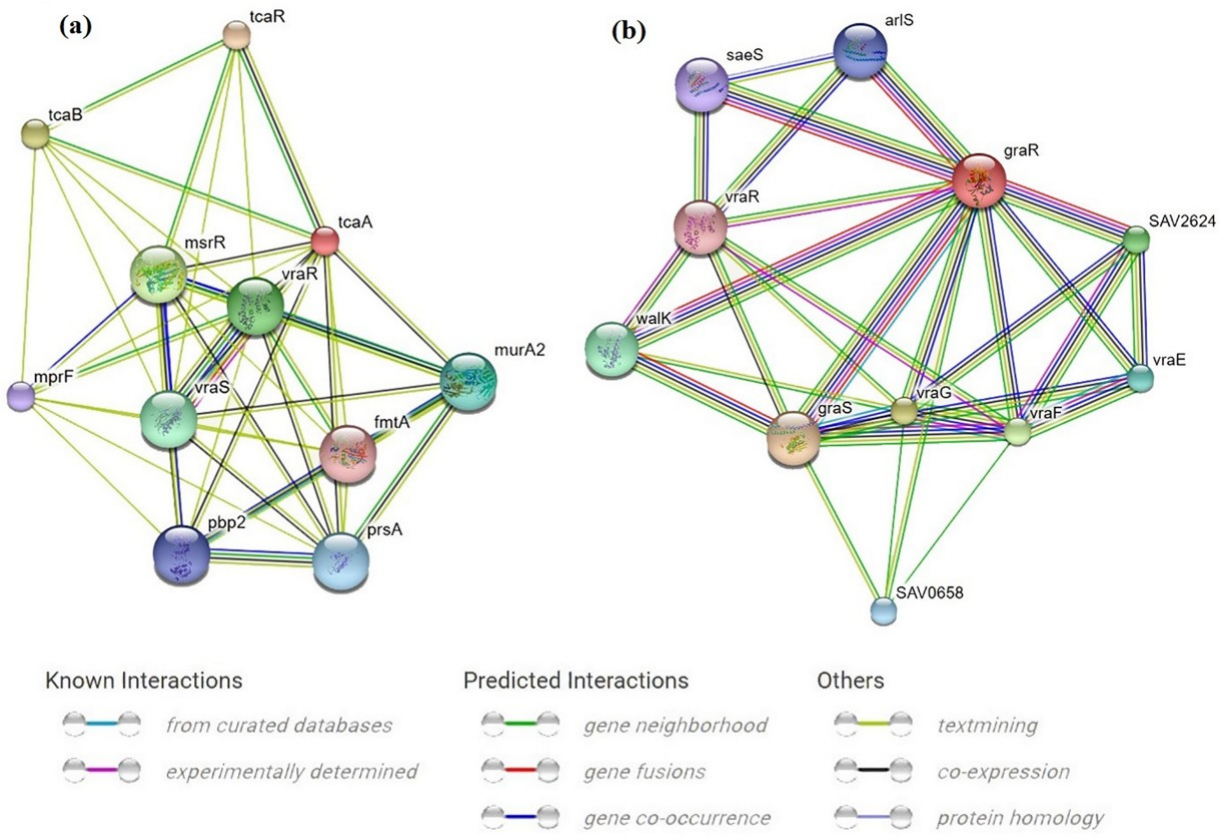
Protein network analysis of selected cell wall biosynthesis regulating genes. a) protein-protein interaction analysis of *tca*A and *tca*B; b) protein-protein interaction analysis of *vra*R, *vra*S, *gra*S, and *gra*R.

The protein-protein interaction analysis predicted that *tca*A showed co-expression with *tca*R, *pbp*2, *vra*R, *vra*S, *msr*R and *mur*A2. Similarly, *wal*K showed co-expression with *gra*R, *gra*S, *wal*K, *vra*R and *sae*S. A functional change induced by the mutations in the *tca*RAB, vraSR, *gra*SR, or *wal*KR TCS, can alter the functioning of other interacting proteins. The *vra*SR TCS was predicted to be directly associated with *pbp*2 (Fig 5). Amino acid substitution induced changes in *vra*SR may influence the expression of *pbp*2.

### Quantitative transcript analysis of *pbp2* and *pbp4*

**Fig 6:**
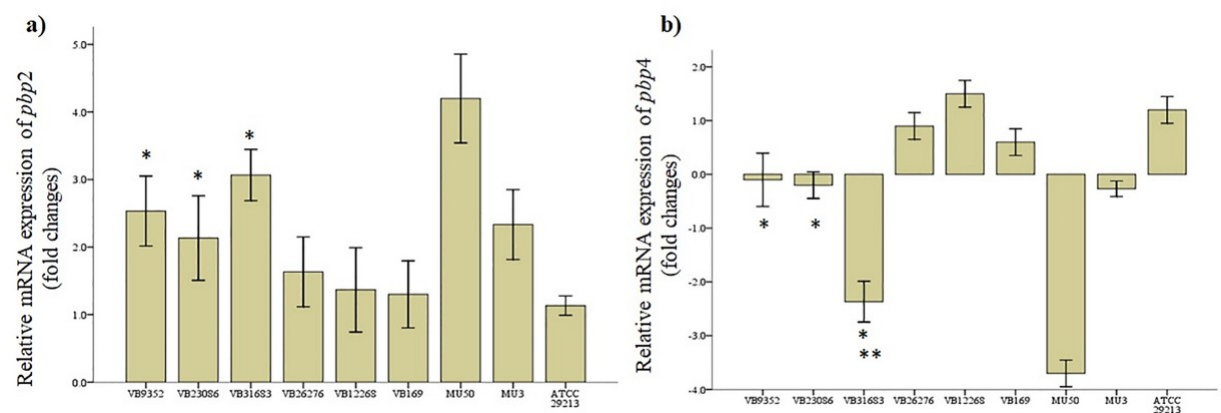
Quantitative transcription of *pbp*2 and *pbp*4 genes among TR-MRSA strains (n = 6). Data are indicated as mean and standard deviation. Error bar represents the standard deviation of three mean values. Upregulation of *pbp*2 and downregulation of *pbp*4 were significant in VB9352, VB23686, and VB31683 compared to *S*. *aureus* ATCC 29213 (marked with a single asterisk, p < 0.01). VB31683 showed significant *pbp*4 downregulation compared to the MU3 strain (marked with a double asterisk, p < 0.01). Expression levels of *pbp*2 were comparable to that of the MU3 strain.

**Fig 7:**
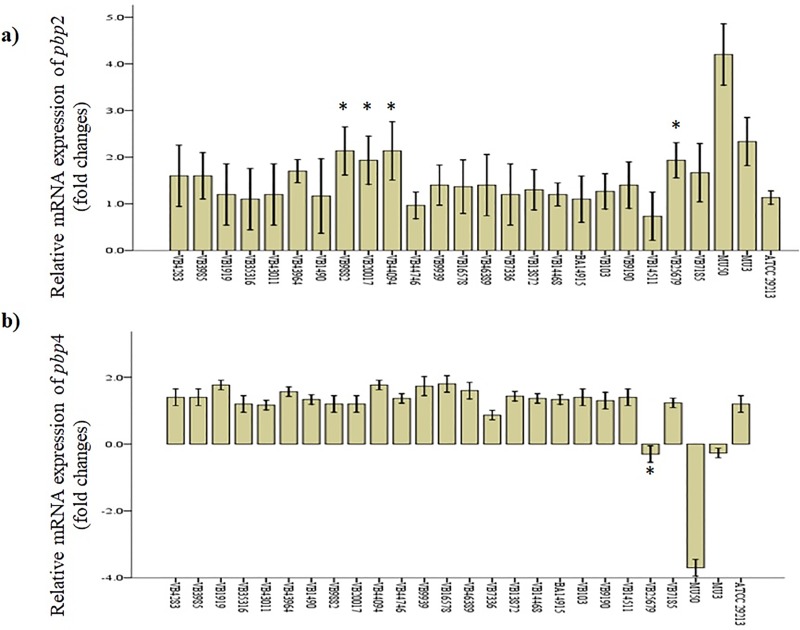
Quantitative transcription of *pbp*2 and *pbp*4 genes in whole-genome sequenced hVISA strains (n = 23). Data are indicated as mean and standard deviation. Error bar represents the standard deviation of three mean values. *pbp*2 upregulation was significant in VB9882, VB20017, VB44094, and VB25679 compared to *S*. *aureus* ATCC 29213 (marked with a single asterisk, p < 0.05). *pbp*4 upregulation was significant in VB25679 compared to *S*. *aureus* ATCC 29213 (marked with a single asterisk, p < 0.05).

Transcriptional changes in *pbp*2 and *pbp*4 were evaluated in both TR-MRSA (n = 6) and hVISA (n = 23) using qRT-PCR. In addition, genomes of TR-MRSA and hVISA were analysed for amino acid substitutions. Eleven distinct substitutions (P285A, T439V, A420V, A557T, T691A, P825A, T489E, H121R, R262C, A172T) in *pbp*2 and seven different substitution (D98E, T253P, E398A, T25A, Q283A, S395C, A409T) in *pbp*4 were observed (S6 Table). In TR-MRSA, *pbp*2 upregulation was found to be significant in VB9352 (2.5-fold), VB23686 (2.2-fold), and VB31683 (3.1-fold), compared to *S*. *aureus* ATCC 29213 (p < 0.01) (Fig 6). Similarly, downregulation of *pbp*4 in VB9352 (0.2-fold), VB23686 (0.2-fold), and VB31683 (2.4-fold) were significant, compared to *S*. *aureus* ATCC 29213 (p < 0.01) (Fig 6). One isolate VB31683 had significant *pbp*4 downregulation compared to the MU3 strain (p < 0.01). Compared to *S*. *aureus* ATCC 29213, *pbp*2 upregulation was found to be significant in four hVISA strains which include VB9882 (2.1-fold), VB20017(1.9-fold), VB44094 (2.1-fold), and VB25679 (1.9-fold) (p < 0.05) (Fig 7). Significant *pbp4* downregulation was observed in only one hVISA (VB25679, 0.3-fold) strain (p <0.01).

Compared to MU3, *pbp*2 upregulation and *pbp*4 downregulation were significant in the TR-MRSA strain VB31683 (p < 0.01). However, none of the tested TR-MRSA strains had significant changes in the transcription of *pbp*2 and *pbp*4, compared to MU50. Similarly, in all the tested hVISA strains, fold changes in *pbp*2 and *pbp*4 transcription were not significant, compared to MU3.

### Accession numbers

All draft genomes used in this study were deposited under DDBJ/ENA/GenBank under the accession numbers as provided in the S7 Table.

## Discussion

In recent years, hVISA and VISA associated vancomycin treatment failure are becoming an increasing clinical challenge. Several studies have reported the occurrence of vancomycin MIC creep in *S*. *aureus* [55–57]. Vancomycin and teicoplanin exhibit antimicrobial activity by binding to D-Ala-D-Ala subunits of the murein monomer. Therefore, cross-resistance can be expected between these antibiotics. The thickening of the cell wall contributes to the development of vancomycin and teicoplanin non-susceptibility in *S*. *aureus*.

The lack of universal resistance markers in hVISA/VISA strains, is a major problem in understanding the genetic mechanism of glycopeptide resistance. The genes *vra*SR, *gra*SR, *wal*KR and *rpo*B have been frequently associated with the development of heterogeneous resistance to vancomycin [8]. The *tca*RAB operon has been reported to contribute to the development of teicoplanin resistance in *S*. *aureus* [58,59]. Here, we examined the presence of mutations in the TCS operon (*tca*RAB, *vra*SR, *vra*T, *gra*SR, *wal*KR, *lyt*SR) and genes (*rpo*B, *clp*, *sae*S, *pho*R, *pbp*2, *pbp*4) in TR-MRSA and hVISA strains. All these loci have been reported as crucial markers for the development of hVISA/VISA strains [8,60].

Studies have reported TR-MRSA infection with a teicoplanin and vancomycin MIC of 16 μg/ml and 2 μg/ml, respectively [61,62]. A meta-analysis showed a pooled prevalence of 6% hVISA and 3% VISA from reports available across the world [11]. In India, the prevalence of hVISA ranges from 5.8% to 6.9% [63,64]. Interestingly, our study documented the presence of 1% TR-MRSA and 12% hVISA amongst MRSA isolates from bloodstream infection. In contrast to previous reports from India, a high prevalence of hVISA was observed in this study.

In this study, we noted novel mutations in important candidate genes *tca*A (D230E, F290S); *tca*B (Y6R, L173M); *vra*S (H6Y, K396R); *vra*R (T24K); *gra*S (T224K); *gra*R (D147E) and *rpo*B (S486L). Mutations in *yvq*F and *vra*SR are reported to be associated with the development of TR-MRSA [21]. However, a similar mutation pattern was not noted in our study. In *S*. *aureus*, *tca*A mutants contribute to the development of VISA [65, 66]. We identified novel mutations in *tca*A and *tca*B. Multiple mutations in *vra*SR loci are frequently associated with hVISA/VISA development [67]. The presence of V15G mutation in *vra*S is associated with increased vancomycin MIC in VISA phenotypes [68]. Notably, in our study, this substitution was identified in hVISA strains. However, a significant rise in vancomycin MIC did not occur in these strains.

Mutation in *gra*S (L26F, I59L, T224I) has not only been reported in hVISA/VISA strains, but also identified in VSSA strains [65,66,69]. In our study, these mutations were noted in both TR-MRSA and hVISA phenotypes, but the significance of these mutations remains unclear. In *gra*R, the D184Q mutation is reported to be strongly associated with the development of hVISA phenotypes [70]. Similarly, in this study, 65% of the tested hVISA strains had D184Q as the predominant substitution.

In *rpo*B, H48IY/N is the predominant loci contributing to the development of dual resistance to rifampicin and vancomycin [68,71]. In our study, all *rpo*B mutations were identified in the rifampicin resistance determining region (RRDR), which spans amino acid residues between positions 463–550. A novel mutation identified in the *rpo*B (S488L) gene of the tested isolate resulted in an increased rifampicin MIC of >32 μg/ml. A large number of distinct mutations were observed, however none of them reliably differentiated TR-MRSA from hVISA strains.

The heterogeneous clonal background could contribute to the diverse mutations observed in this study. Most of the reported hVISA/VISA strains belong to ST5 (clonal complex, CC5) or ST239 (CC8) [8]. Remarkably, in our study, sixteen different STs belonged to eight different CCs. A significant association was not noted between STs and amino acid substitutions. However, mutations in *tca*A (D230E) and *gra*S (T224I) were commonly seen in ST772 MRSA strains, whilst a D148Q mutation in *gra*R was seen in diverse STs. This reflects the adaptation of different MRSA genotypes to develop TR-MRSA/hVISA phenotypes. This is the first study to report TR-MRSA and hVISA strains belonging to ST772 and ST22.

A change in the expression of *pbp*2 and *pbp*4 leads to a thickened cell wall in *S*. *aureus* with reduced vancomycin susceptibility [72]. Upregulation of *pbp*2 promotes cell wall synthesis, and downregulation of *pbp*4 results in decreased murein cross-linking [73]. These substantial changes may result in increased production of D-Ala-D-Ala subunits which trap most of the vancomycin molecule. In our study, significant *pbp*2 upregulation was noted in TR-MRSA (MIC 16 or 32 μg/ml) and four hVISA strains. However, none of the tested isolates showed significant *pbp*4 downregulation. This could be due to differences in the mutation occurring in co-expressed genes which are involved in cell wall synthesis.

Studies have reported the potential association between *agr* dysfunction and hVISA/VISA [74]. Harigaya *et al*. reported *agr* dysfunction in hVISA strains was five times more than that of VSSA strains [75]. A study from India reported 82.8% of hVISA isolates carried *agr* group I loci. Similarly, in this study, the majority of tested strains belong to *agr* group I.

A limitation of our study is that the genetic effects of the novel mutation on vancomycin susceptibility were not investigated.

## Conclusion

In conclusion, we describe the presence of TR-MRSA and hVISAs isolated from bloodstream infection. A significantly higher proportion of hVISA isolates were identified among MRSA isolates with vancomycin MIC of ≥ 1 μg/ml. A large number of mutations were identified in the candidate genes. This indicates the complex evolutionary pathway involved in the development of TR-MRSA and hVISA. However, none of the markers were reliable in differentiating hVISA from TR-MRSA phenotypes. Considerably significant upregulation of *pbp*2 was noted in the strains with high teicoplanin MICs of 16 or 32 μg/ml. Mutated *tca*RAB, *vra*SR, *gra*SR, and *rpo*B *may* influence the transcription of the cell wall biosynthesis gene.

## Supporting information

S1 Fig Distribution of vancomycin and teicoplanin minimum inhibitory concentration (MIC) among MRSA isolated from bloodstream infection. (TIF)

S1 Table Population analysis profile- area under curve (PAP-AUC) analysis of hVISA isolated from bloodstream infection. (DOCX)

S2 Table Mutation pattern observed in the different sequence types of hVISA strains. (DOCX)

S3 Table Difference in the mutation observed between TR-MRSA and hVISA strains. (DOCX)

S4 Table Triton X-100 induced autolysis rate and doubling time observed in teicoplanin-resistant MRSA (TR-MRSA) strains. (DOCX)

S5 Table Triton X-100 induced autolysis rate and doubling time observed in heterogeneous resistant vancomycin intermediate *S*. *aureus* (hVISA) strains. (DOCX)

S6 Table Mutations identified in the *pbp*2 and *pbp*4 genes of TR-MRSA and hVISA strains. (DOCX)

S7 Table Accession numbers of TR-MRSA and hVISA genome and their sequence types. (DOCX)

## References

[1] Melzer M, Eykyn SJ, Gransden WR, Chinn S. Is methicillin-resistant *Staphylococcus aureus* more virulent than methicillin-susceptible *S*. *aureus*? A comparative cohort study of British patients with nosocomial infection and bacteremia. Clin Infect Dis. 2003;37(11):1453–1460. 10.1086/379321 14614667

[2] Clinical and Laboratory Standards Institute (CLSI). Performance standards for antimicrobial susceptibility testing; 28th informational supplement CLSI document M100-S28; 2018, Clinical and Laboratory Standards Institute, Wayne, PA.

[3] European Committee on Antimicrobial Susceptibility Testing (EUCAST). Breakpoint tables for interpretation of MICs and zone diameters. 2018, version 8.0. http://www.eucast.org/fileadmin/src/media/PDFs/EUCAST_files/Breakpoint_tables/v_8.0_Breakpoint_Tables.pdf

[4] Soriano A, Marco F, Martínez JA, Pisos E, Almela M, Dimova VP et al Influence of vancomycin minimum inhibitory concentration on the treatment of methicillin-resistant Staphylococcus aureus bacteremia. Clin Infect Dis. 2008;46(2):193–200. 10.1086/524667 18171250

[5] Song KH, Kim M, Kim CJ, Cho JE, Choi YJ, Park JS et al Impact of Vancomycin MIC on Treatment Outcomes in Invasive *Staphylococcus aureus* Infections. Antimicrob Agents Chemother. 2017;61(3). pii: e01845-16. 10.1128/AAC.01845-16 PMC5328555 27956430

[6] Chang HJ, Hsu PC, Yang CC, Siu LK, Kuo AJ, Chia JH, et al Influence of teicoplanin MICs on treatment outcomes among patients with teicoplanin-treated methicillin-resistant *Staphylococcus aureus* bacteraemia: a hospital-based retrospective study. J Antimicrob Chemother. 2012;67(3):736–741. 10.1093/jac/dkr531 22169187

[7] Chen KY, Chang HJ, Hsu PC, Yang CC, Chia JH, Wu TL et al Relationship of teicoplanin MICs to treatment failure in teicoplanin-treated patients with methicillin-resistant *Staphylococcus aureus* pneumonia. J Microbiol Immunol Infect. 2013;46(3):210–6. 10.1016/j.jmii.2012.06.010 22999099

[8] Howden BP, Davies JK, Johnson PD, Stinear TP, Grayson ML. Reduced vancomycin susceptibility in *Staphylococcus aureus*, including vancomycin-intermediate and heterogeneous vancomycin-intermediate strains: resistance mechanisms, laboratory detection, and clinical implications. Clin Microbiol Rev. 2010;23(1):99–139. 10.1128/CMR.00042-09 20065327 PMC2806658

[9] Cepeda J, Hayman S, Whitehouse T, Kibbler CC, Livermore D, Singer M. Teicoplanin resistance in methicillin-resistant *Staphylococcus aureus* in an intensive care unit. J Antimicrob Chemother. 2003;52(3):533–4. 10.1093/jac/dkg369 12888581

[10] Gomes DM, Ward KE, LaPlante KL. Clinical implications of vancomycin heteroresistant and intermediately susceptible *Staphylococcus aureus*. Pharmacotherapy. 2015;35(4):424–432. 10.1002/phar.1577 25884530

[11] Zhang S, Sun X, Chang W, Dai Y, Ma X. Systematic Review and Meta-Analysis of the Epidemiology of Vancomycin-Intermediate and Heterogeneous Vancomycin-Intermediate *Staphylococcus aureus* Isolates. 2015;PLoS One.10(8):e0136082 10.1371/journal.pone.0136082 26287490 PMC4546009

[12] van Hal SJ, Jones M, Gosbell IB, Paterson DL. Vancomycin heteroresistance is associated with reduced mortality in ST239 methicillin-resistant *Staphylococcus aureus* blood stream infections. PLoS One. 2011;6(6):e21217 10.1371/journal.pone.0021217 21713004 PMC3119693

[13] Casapao AM, Leonard SN, Davis SL, Lodise TP, Patel N, Goff DA et al Clinical Outcomes in Patients with Heterogeneous Vancomycin-Intermediate *Staphylococcus aureus* Bloodstream Infection. Antimicrob Agents Chemother. 2013;57(9):4252–4259. 10.1128/AAC.00380-13 23796929 PMC3754327

[14] Dhand A, Sakoulas G. Reduced vancomycin susceptibility among clinical *Staphylococcus aureus* isolates ('the MIC Creep'): implications for therapy. F1000 Med Rep. 2014;4:4. 10.3410/M4-4 PMC3270590 22312414

[15] Hiramatsu K. Vancomycin-resistant *Staphylococcus aureus*: a new model of antibiotic resistance. Lancet Infect Dis. 2001;1(3):147–155. 10.1016/S1473-3099(01)00091-3 11871491

[16] Clinical and Laboratory Standards Institute (CLSI). Methods for dilution of antimicrobial susceptibility tests for bacteria that grow aerobically; approved standard, 9th ed. Document M07-A9. 2012; Clinical and Laboratory Standards Institute, Wayne, PA.

[17] Deresinski S. The multiple paths to heteroresistance and intermediate resistance to vancomycin in *Staphylococcus aureus*. J Infect Dis. 2013;208(1):7–9. 10.1093/infdis/jit136 23539742

[18] Xu J, Pang L, Ma XX, Hu J, Tian Y, Yang YL et al Phenotypic and Molecular Characterisation of *Staphylococcus aureus* with Reduced Vancomycin Susceptibility Derivated in Vitro. Open Med (Wars). 2018;13:475–486. 30426085 10.1515/med-2018-0071 PMC6227741

[19] Cui L, Iwamoto A, Lian JQ, Neoh HM, Maruyama T, Horikawa Y, Hiramatsu K. Novel mechanism of antibiotic resistance originating in vancomycin-intermediate *Staphylococcus aureus*. Antimicrob Agents Chemother. 2006;50(2):428–38. 10.1128/AAC.50.2.428-438.2006 16436693 PMC1366884

[20] Boyle-Vavra S, Yin S, Jo DS, Montgomery CP, Daum RS. VraT/YvqF is required for methicillin resistance and activation of the VraSR regulon in *Staphylococcus aureus*. Antimicrob Agents Chemother. 2013;57(1):83–95. 10.1128/AAC.01651-12 23070169 PMC3535960

[21] Elsaghier AA, Aucken HM, Hamilton-Miller JM, Shaw S, Kibbler CC. Resistance to teicoplanin developing during treatment of methicillin-resistant *Staphylococcus aureus* infection. J Antimicrob Chemother. 2002;49(2):423–4. 10.1093/jac/49.2.423 11815594

[22] Szymanek-Majchrzak K, Mlynarczyk A, Mlynarczyk G. Characteristics of glycopeptide-resistant *Staphylococcus aureus* strains isolated from inpatients of three teaching hospitals in Warsaw, Poland. Antimicrob Resist Infect Control. 2018;7:105 10.1186/s13756-018-0397-y 30181870 PMC6114487

[23] Kato Y, Suzuki T, Ida T, Maebashi K. Genetic changes associated with glycopeptide resistance in *Staphylococcus aureus*: predominance of amino acid substitutions in YvqF/VraSR. J Antimicrob Chemother. 2010;65(1):37–45. 10.1093/jac/dkp394 19889788 PMC2800785

[24] McCallum N, Berger-Bächi B, Senn MM. Regulation of antibiotic resistance in *Staphylococcus aureus*. Int J Med Microbiol. 2010;300(2–3):118–129. 10.1016/j.ijmm.2009.08.015 19800843

[25] Maki H, McCallum N, Bischoff M, Wada A, Berger-Bächi B. tcaA inactivation increases glycopeptide resistance in *Staphylococcus aureus*. Antimicrob Agents Chemother. 2004;48(6):1953–9. 10.1128/AAC.48.6.1953-1959.2004 15155184 PMC415614

[26] Brandenberger M, Tschierske M, Giachino P, Wada A, Berger-Bächi B. Inactivation of a novel three-cistronic operon tcaR-tcaA-tcaB increases teicoplanin resistance in *Staphylococcus aureus*. Biochim Biophys Acta. 2000;1523(2–3):135–9. 10.1016/s0304-4165(00)00133-1 11042376

[27] Wootton M, Macgowan AP, Walsh TR. Expression of tcaA and mprF and glycopeptide resistance in clinical glycopeptide-intermediate *Staphylococcus aureus* (GISA) and heteroGISA strains. Biochim Biophys Acta. 2005;1726(3):326–7. 10.1016/j.bbagen.2005.09.002 16213099

[28] Liu C, Chambers HF. *Staphylococcus aureus* with heterogeneous resistance to vancomycin: epidemiology, clinical significance, and critical assessment of diagnostic methods. Antimicrob Agents Chemother. 2003;47(10):3040–5. 10.1128/AAC.47.10.3040-3045.2003 14506006 PMC201119

[29] Brunet F, Vedel G, Dreyfus F, Vaxelaire JF, Giraud T, Schremmer B et al Failure of teicoplanin therapy in two neutropenic patients with staphylococcal septicemia who recovered after administration of vancomycin. Eur J Clin Microbiol Infect Dis. 1990;9(2):145–7. 10.1007/bf01963643 2138543

[30] Hiramatsu K. Vancomycin-resistant *Staphylococcus aureus*: a new model of antibiotic resistance. Lancet Infect Dis. 2001;1(3):147–55. 10.1016/S1473-3099(01)00091-3 11871491

[31] Sieradzki K, Villari P, Tomasz A. Decreased susceptibilities to teicoplanin and vancomycin among coagulase-negative methicillin-resistant clinical isolates of staphylococci. Antimicrob Agents Chemother. 1998;42(1):100–7 9449268 10.1128/aac.42.1.100 PMC105463

[32] Tille P. M., & Forbes B. A. Bailey & Scott's diagnostic microbiology (Thirteenth edition). 2014;St. Louis, Missouri: Elsevier.

[33] Khatib R, Riederer K, Sharma M, Shemes S, Iyer SP, Szpunar S. Screening for Intermediately Vancomycin-Susceptible and Vancomycin-Heteroresistant *Staphylococcus aureus* by Use of Vancomycin-Supplemented Brain Heart Infusion Agar Biplates: Defining Growth Interpretation Criteria Based on Gold Standard Confirmation. J Clin Microbiol. 2015;53(11):3543–6. 10.1128/JCM.01620-15 26311860 PMC4609695

[34] Satola SW, Farley MM, Anderson KF, Patel JB. Comparison of detection methods for heteroresistant vancomycin-intermediate *Staphylococcus aureus*, with the population analysis profile method as the reference method. J Clin Microbiol. 2011;49(1):177–183. 10.1128/JCM.01128-10 21048008 PMC3020420

[35] Wootton M, Howe RA, Hillman R, Walsh TR, Bennett PM, MacGowan AP. A modified population analysis profile (PAP) method to detect hetero-resistance to vancomycin in *Staphylococcus aureus* in a UK hospital. J Antimicrob Chemother. 2001;47(4):399–403. 10.1093/jac/47.4.399 11266410

[36] Rodriguez CA, Agudelo M, Zuluaga AF, Vesga O. Generic vancomycin enriches resistant subpopulations of *Staphylococcus aureus* after exposure in a neutropenic mouse thigh infection model. Antimicrob Agents Chemother. 2012;56(1):243–7. 10.1128/AAC.05129-11 22064531 PMC3256022

[37] Chen CJ, Huang YC, Chiu CH. Multiple pathways of cross-resistance to glycopeptides and daptomycin in persistent MRSA bacteraemia. J Antimicrob Chemother. 2015;70(11):2965–72 10.1093/jac/dkv225 26216581

[38] Goudarzi M, Seyedjavadi SS, Nasiri MJ, Goudarzi H, Sajadi Nia R, Dabiri H. Molecular characteristics of methicillin-resistant *Staphylococcus aureus* (MRSA) strains isolated from patients with bacteremia based on MLST, SCCmec, spa, and agr locus types analysis. Microb Pathog. 2017;104:328–335. 10.1016/j.micpath.2017.01.055 28159661

[39] Zhang K, McClure JA, Elsayed S, Louie T, Conly JM. Novel multiplex PCR assay for characterization and concomitant subtyping of staphylococcal cassette chromosome mec types I to V in methicillin-resistant *Staphylococcus aureus*. J Clin Microbiol. 2005;43(10):5026–5033. 10.1128/JCM.43.10.5026-5033.2005 16207957 PMC1248471

[40] Ghaznavi-Rad E, Nor Shamsudin M, Sekawi Z, van Belkum A, Neela V. A simplified multiplex PCR assay for fast and easy discrimination of globally distributed staphylococcal cassette chromosome mec types in meticillin-resistant *Staphylococcus aureus*. J Med Microbiol. 2010;59(Pt 10):1135–9. 10.1099/jmm.0.021956-0 20616192

[41] Harmsen D, Claus H, Witte W, Rothgänger J, Claus H, Turnwald D et al Typing of methicillin-resistant *Staphylococcus aureus* in a university hospital setting by using novel software for spa repeat determination and database management. J Clin Microbiol. 2003;41(12):5442–8. 10.1128/JCM.41.12.5442-5448.2003 14662923 PMC309029

[42] Enright MC, Day NP, Davies CE, Peacock SJ, Spratt BG. Multilocus sequence typing for characterization of methicillin-resistant and methicillin-susceptible clones of *Staphylococcus aureus*. J Clin Microbiol. 2000;38(3):1008–1015. 10698988 10.1128/jcm.38.3.1008-1015.2000 PMC86325

[43] Ribeiro-Gonçalves B, Francisco AP, Vaz C, Ramirez M, Carriço JA. PHYLOViZ Online: web-based tool for visualization, phylogenetic inference, analysis and sharing of minimum spanning trees. Nucleic Acids Res. 2016;44(W1):W246–251. 10.1093/nar/gkw359 27131357 PMC4987911

[44] Wattam A.R., Abraham D., Dalay O., Disz T.L., Driscoll T., Gabbard J.L., Gillespie J.J. et al PATRIC, the bacterial bioinformatics database and analysis resource. Nucleic Acids Res. 2013;42:D581–591. 10.1093/nar/gkt1099 24225323 PMC3965095

[45] Aziz RK, Bartels D, Best AA, DeJongh M, Disz T, Edwards RA et al The RAST Server: rapid annotations using subsystems technology. BMC genomics. 2008;9(1):75 18261238 10.1186/1471-2164-9-75 PMC2265698

[46] Overbeek R, Olson R, Pusch GD, Olsen GJ, Davis JJ, Disz T et al The SEED and the Rapid Annotation of microbial genomes using Subsystems Technology (RAST). Nucleic acids research. 2013;42(D1):D206–214 24293654 10.1093/nar/gkt1226 PMC3965101

[47] Brettin T, Davis JJ, Disz T, Edwards RA, Gerdes S, Olsen GJ et al RASTtk: a modular and extensible implementation of the RAST algorithm for building custom annotation pipelines and annotating batches of genomes. Scientific reports. 2015;5:8365 10.1038/srep08365 25666585 PMC4322359

[48] Ng PC, Henikoff S. Predicting deleterious amino acid substitutions. Genome Res. 2001;11(5):863–74. 10.1101/gr.176601 11337480 PMC311071

[49] Huang T, Wang P, Ye ZQ, Xu H, He Z, Feng KY et al Prediction of deleterious non-synonymous SNPs based on protein interaction network and hybrid properties. PLoS One. 2010;5(7):e11900 10.1371/journal.pone.0011900 20689580 PMC2912763

[50] Shannon P, Markiel A, Ozier O, Baliga NS, Wang JT, Ramage D et al Cytoscape: A Software Environment for Integrated Models of Biomolecular Interaction Networks. Genome Research. 2003;13(11), 2498–2504. 10.1101/gr.1239303 14597658 PMC403769

[51] Szklarczyk D, Franceschini A, Wyder S, Forslund K, Heller D, Huerta-Cepas J et al STRING v10: protein-protein interaction networks, integrated over the tree of life. Nucleic Acids Research. 2015; 43(Database issue), D447–52. 10.1093/nar/gku1003 25352553 PMC4383874

[52] Renzoni A, Barras C, François P, Charbonnier Y, Huggler E, Garzoni C et al Transcriptomic and functional analysis of an autolysis-deficient, teicoplanin-resistant derivative of methicillin-resistant *Staphylococcus aureus*. Antimicrob Agents Chemother. 2006;50(9):3048–3061. 10.1128/AAC.00113-06 16940101 PMC1563528

[53] Livak KJ, Schmittgen TD. Analysis of relative gene expression data using real-time quantitative PCR and the 2(-Delta Delta C(T)) Method. Methods. 2001;25(4):402–8. 10.1006/meth.2001.1262 11846609

[54] Vaudaux P, Francois P, Bisognano C, Kelley WL, Lew DP, Schrenzel J et al Increased expression of clumping factor and fibronectin-binding proteins by hemB mutants of *Staphylococcus aureus* expressing small colony variant phenotypes. Infect Immun. 2002;70(10):5428–5437. 10.1128/IAI.70.10.5428-5437.2002 12228267 PMC128368

[55] Lodise TP, Graves J, Evans A, Graffunder E, Helmecke M, Lomaestro BM et al Relationship between vancomycin MIC and failure among patients with methicillin-resistant *Staphylococcus aureus* bacteremia treated with vancomycin. Antimicrob Agents Chemother. 2008;52(9):3315–3320. 10.1128/AAC.00113-08 18591266 PMC2533486

[56] Sakoulas G, Moise-Broder PA, Schentag J, Forrest A, Moellering RC Jr, Eliopoulos GM. Relationship of MIC and bactericidal activity to efficacy of vancomycin for treatment of methicillin-resistant *Staphylococcus aureus* bacteremia. J Clin Microbiol. 2004;42(6):2398–2402. 10.1128/JCM.42.6.2398-2402.2004 15184410 PMC427878

[57] Yeh YC, Yeh KM, Lin TY, Chiu SK, Yang YS, Wang YC et al Impact of vancomycin MIC creep on patients with methicillin-resistant *Staphylococcus aureus* bacteremia. J Microbiol Immunol Infect. 2012;45(3):214–220. 10.1016/j.jmii.2011.11.006 22571999

[58] Hiramatsu K, Kayayama Y, Matsuo M, Aiba Y, Saito M, Hishinuma T et al Vancomycin-intermediate resistance in *Staphylococcus aureus*. J Glob Antimicrob Resist. 2014;2(4):213–224. 10.1016/j.jgar.2014.04.006 27873679

[59] Song JH, Hiramatsu K, Suh JY, Ko KS, Ito T, Kapi M et al Asian Network for Surveillance of Resistant Pathogens Study Group. Emergence in Asian countries of *Staphylococcus aureus* with reduced susceptibility to vancomycin. Antimicrob Agents Chemother. 2004;48(12):4926–8. 10.1128/AAC.48.12.4926-4928.2004 15561884 PMC529235

[60] Chaudhari CN, Tandel K, Grover N, Sen S, Bhatt P, Sahni AK et al Heterogeneous vancomycin-intermediate among methicillin resistant *Staphylococcus aureus*. Med J Armed Forces India. 2015;71(1):15–8. 10.1016/j.mjafi.2014.03.008 25609857 PMC4297820

[61] Singh A, Prasad KN, Misra R, Rahman M, Singh SK, Rai RP et al Increasing Trend of Heterogeneous Vancomycin Intermediate *Staphylococcus aureus* in a Tertiary Care Center of Northern India. Microb Drug Resist. 2015;21(5):545–50. 10.1089/mdr.2015.0004 26430942

[62] Wang Y, Li X, Jiang L, Han W, Xie X, Jin Y et al Novel Mutation Sites in the Development of Vancomycin- Intermediate Resistance in *Staphylococcus aureus*. Front Microbiol. 2017;7:2163 10.3389/fmicb.2016.02163 28119680 PMC5222870

[63] Doddangoudar VC, Boost MV, Tsang DN, O'Donoghue MM. Tracking changes in the vraSR and graSR two component regulatory systems during the development and loss of vancomycin non-susceptibility in a clinical isolate. Clin Microbiol Infect. 2011;17(8):1268–1272. 10.1111/j.1469-0691.2011.03463.x 21375655

[64] Alam MT, Petit RA 3rd, Crispell EK, Thornton TA, Conneely KN, Jiang Y et al Dissecting vancomycin-intermediate resistance in *Staphylococcus aureus* using genome-wide association. Genome Biol Evol. 2014;6(5):1174–1185. 10.1093/gbe/evu092 24787619 PMC4040999

[65] Yoo JI, Kim JW, Kang GS, Kim HS, Yoo JS, Lee YS. Prevalence of amino acid changes in the yvqF, vraSR, graSR, and tcaRAB genes from vancomycin intermediate resistant *Staphylococcus aureus*. J Microbiol. 2013;51(2):160–5. 10.1007/s12275-013-3088-7 23625215

[66] Lin LC, Chang SC, Ge MC, Liu TP, Lu JJ. Novel single-nucleotide variations associated with vancomycin resistance in vancomycin-intermediate *Staphylococcus aureus*. Infect Drug Resist. 2018;11:113–123. 10.2147/IDR.S148335 29403293 PMC5783010

[67] Hafer C, Lin Y, Kornblum J, Lowy FD, Uhlemann AC. Contribution of selected gene mutations to resistance in clinical isolates of vancomycin-intermediate *Staphylococcus aureus*. Antimicrob Agents Chemother. 2012;56(11):5845–5851. 10.1128/AAC.01139-12 22948864 PMC3486570

[68] Watanabe Y, Cui L, Katayama Y, Kozue K, Hiramatsu K. Impact of rpoB mutations on reduced vancomycin susceptibility in *Staphylococcus aureus*. J Clin Microbiol. 2011;49(7):2680–4. 10.1128/JCM.02144-10 21525224 PMC3147882

[69] Roch M, Clair P, Renzoni A, Reverdy ME, Dauwalder O, Bes M. Exposure of *Staphylococcus aureus* to subinhibitory concentrations of β-lactam antibiotics induces heterogeneous vancomycin-intermediate *Staphylococcus aureus*. Antimicrob Agents Chemother. 2014;58(9):5306–5314. 10.1128/AAC.02574-14 24957836 PMC4135816

[70] Hu Q, Peng H, Rao X. Molecular Events for Promotion of Vancomycin Resistance in Vancomycin Intermediate *Staphylococcus aureus*. Front Microbiol. 2016;7:1601 10.3389/fmicb.2016.01601 27790199 PMC5062060

[71] Gao W, Cameron DR, Davies JK, Kostoulias X, Stepnell J, Tuck KL. The RpoB H₄₈₁Y rifampicin resistance mutation and an active stringent response reduce virulence and increase resistance to innate immune responses in *Staphylococcus aureus*. J Infect Dis. 2013;207(6):929–939. 10.1093/infdis/jis772 23255563 PMC3633451

[72] Sieradzki K, Tomasz A. Alterations of cell wall structure and metabolism accompany reduced susceptibility to vancomycin in an isogenic series of clinical isolates of *Staphylococcus aureus*. J Bacteriol. 2003;185(24):7103–7110. 10.1128/JB.185.24.7103-7110.2003 14645269 PMC296238

[73] Gardete S, Tomasz A. Mechanisms of vancomycin resistance in *Staphylococcus aureus*. J Clin Invest. 2014;124(7):2836–2840 10.1172/JCI68834 24983424 PMC4071404

[74] Park C, Shin NY, Byun JH, Shin HH, Kwon EY, Choi SM. Downregulation of RNAIII in vancomycin-intermediate *Staphylococcus aureus* strains regardless of the presence of agr mutation. J Med Microbiol. 2012;61(Pt 3):345–352. 10.1099/jmm.0.035204-0 22016559

[75] Harigaya Y, Ngo D, Lesse AJ, Huang V, Tsuji BT. Characterization of heterogeneous vancomycin-intermediate resistance, MIC and accessory gene regulator (agr) dysfunction among clinical bloodstream isolates of *Staphyloccocus aureus*. BMC Infect Dis. 2011;11:287 10.1186/1471-2334-11-287 22026752 PMC3215976

