# Supplementary material for: Genomic insights on heterogeneous resistance to vancomycin and teicoplanin in Methicillin-resistant Staphylococcus aureus: A first report from South India
Source: PLoS One. 2019 Dec 30;14(12):e0227009. doi: 10.1371/journal.pone.0227009 (PMC6936811; doi:10.1371/journal.pone.0227009)
Supplement: S1 Table — (DOCX) [file pone.0227009.s002.docx]

**S1 Table.**

| **S. No** | **Micro ID** | **Year** | **Van MIC (µg/ml)** | **PAP-AUC ratio** |
| --- | --- | --- | --- | --- |
| 1 | B6832 | 2013 | 1 | 0.92 |
| 2 | B38761 | 2013 | 1 | 1.11 |
| 3 | B44746 | 2013 | 1 | 1.26 |
| 4 | B6907 | 2013 | 1 | 0.98 |
| 5 | B30101 | 2014 | 1 | 1.01 |
| 6 | FF1489 | 2014 | 1 | 0.9 |
| 7 | FF2016 | 2014 | 1 | 1 |
| 8 | FF507 | 2014 | 1 | 1.17 |
| 9 | B13333 | 2014 | 1 | 0.96 |
| 10 | B9882 | 2015 | 1.5 | 1.03 |
| 11 | BA43964 | 2015 | 1 | 1.02 |
| 12 | BA46389 | 2015 | 1 | 1.03 |
| 13 | BA43011 | 2015 | 1 | 1.28 |
| 14 | CS1919 | 2015 | 1 | 0.92 |
| 15 | FF1490 | 2015 | 1 | 1.01 |
| 16 | B20017 | 2015 | 1.5 | 0.96 |
| 17 | BA44094 | 2015 | 1.5 | 1.23 |
| 18 | B35316 | 2015 | 1 | 0.96 |
| 19 | B3985 | 2016 | 1 | 1.14 |
| 20 | BA15100 | 2016 | 1 | 1.19 |
| 21 | BP3859 | 2016 | 1 | 1.1 |
| 22 | B7185 | 2016 | 1 | 0.9 |
| 23 | BA32883 | 2016 | 1 | 0.98 |
| 24 | BA33052 | 2016 | 1 | 0.95 |
| 25 | BA16104 | 2016 | 1 | 0.91 |
| 26 | BA21032 | 2016 | 1 | 0.9 |
| 27 | BA22553 | 2016 | 0.5 | 0.92 |
| 28 | BA13872 | 2017 | 1 | 0.95 |
| 29 | B14511 | 2017 | 1 | 0.92 |
| 30 | BA11894 | 2017 | 1 | 1.11 |
| 31 | BA31796 | 2017 | 1 | 0.92 |
| 32 | B4283 | 2017 | 1 | 1.25 |
| 33 | BA6415 | 2017 | 0.5 | 1.06 |
| 34 | BA3792 | 2017 | 0.5 | 1.09 |
| 35 | BP3820 | 2017 | 1 | 0.93 |
| 36 | BP834 | 2017 | 0.5 | 1.04 |
| 37 | BA5031 | 2017 | 0.5 | 1.25 |
| 38 | BA5309 | 2017 | 0.5 | 1.04 |
| 39 | BA10785 | 2017 | 0.5 | 0.93 |
| 40 | BA14915 | 2017 | 1 | 1.03 |
| 41 | BP5187 | 2017 | 0.5 | 1.13 |
| 42 | BA1801 | 2017 | 0.5 | 0.98 |
| 43 | BP13568 | 2017 | 1 | 1.13 |
| 44 | BA21900 | 2017 | 1 | 1.09 |
| 45 | BA24325 | 2017 | 0.5 | 1.13 |
| 46 | BA12804 | 2017 | 0.5 | 1.09 |
| 47 | BA24023 | 2017 | 0.5 | 0.95 |
| 48 | BA33868 | 2017 | 0.5 | 0.92 |
| 49 | BA103 | 2018 | 1 | 1 |
| 50 | BA14468 | 2018 | 1 | 0.97 |
| 51 | BP7336 | 2018 | 1 | 0.99 |
| 52 | BA5275 | 2018 | 0.5 | 0.9 |
| 53 | BA20860 | 2018 | 0.5 | 0.94 |
| 54 | B9939 | 2015 | 1 | 0.91 |
| 55 | B16578 | 2015 | 1 | 0.96 |
| 56 | B9190 | 2015 | 1 | 1.1 |
| 57 | BA35739 | 2017 | 1 | 1.23 |
| 58 | BA25679 | 2017 | 2 | 1.27 |

* Van - Vancomycin
