# Supplementary material for: Genomic insights on heterogeneous resistance to vancomycin and teicoplanin in Methicillin-resistant Staphylococcus aureus: A first report from South India
Source: PLoS One. 2019 Dec 30;14(12):e0227009. doi: 10.1371/journal.pone.0227009 (PMC6936811; doi:10.1371/journal.pone.0227009)
Supplement: S2 Table — (DOCX) [file pone.0227009.s003.docx]

**S2 Table.**

| **Gene** | **Amino acid substitution Pattern** | **No. of Isolates** | **ST Type** |
| --- | --- | --- | --- |
| *tca*A | D230E  F290S  D230E, Y237H  L218P, G312D | 7  5  1  1 | ST772(7)  ST22(3), ST2371(2)  ST772(1)  ST6(1) |
| *tca*B | L173M  K396R | 1  1 | ST1482(1)  ST6(1) |
| *vra*S | V15G  H6Y, K396R | 2  1 | ST239(2)  ST6(1) |
| *vra*R | T24K | 2 | ST772(2) |
| *gra*S | T224I  L26F, I59L, R232K  L26F, I59L  L26F, T224I  L26F, I59L, T224I | 12  1  1  2  2 | ST772(7), ST72(1), ST580(1), ST1482(1), ST1(1), ST2371(1)  ST239(1)  ST239(1)  ST1290(1), ST6(1)  ST368(1), ST239(1) |
| *gra*R | D148Q  D147E, D148 | 15  1 | ST22(4), ST772(4), ST2371(2), ST239(3), ST1482(1), ST368(1)  ST580(1) |
| *rpo*B | N474S  H481N, L466S  S486L | 1  1  1 | ST2371(1)  ST239(1)  ST1(1) |
| *lyt*R | G122D  T118N, N125S  E31D | 3  1  1 | ST239(3)  ST6(1)  ST72(1) |
| *sae*S | I340M  L203V | 3  3 | ST22(2), ST2371(1)  ST239(2), ST368(1) |
| *mpr*F | T635I, E709D  T635I | 1  2 | ST22(1)  ST2371(2) |
