# Supplementary material for: Genomic insights on heterogeneous resistance to vancomycin and teicoplanin in Methicillin-resistant Staphylococcus aureus: A first report from South India
Source: PLoS One. 2019 Dec 30;14(12):e0227009. doi: 10.1371/journal.pone.0227009 (PMC6936811; doi:10.1371/journal.pone.0227009)
Supplement: S3 Table — (DOCX) [file pone.0227009.s004.docx]

**S3 Table.**

| **Genes** | **Amino acid substitution (SIFT score)** | **Reduced teicoplanin susceptible MRSA** | **hVISA** |
| --- | --- | --- | --- |
| *tca*A | D230E (1.0) | + | + |
|  | Y237H (0.63) | + | + |
|  | F290S (0.71) | + | + |
| *tca*B | Y6R (0.001) | + | - |
|  | H6Y (1.0) | + | - |
|  | L173M (0.4) | - | + |
|  | K396R (0.001) | - | + |
| *vra*S | V15G (0.001) | - | + |
|  | H6Y (1.0) | - | + |
|  | K396R (0.001) | - | + |
| *vra*R | T24K (0.5) | - | + |
| *gra*S | L26F (0.5) | - | + |
|  | I59L (1.0) | - | + |
|  | T224I (0.4) | + | + |
|  | T224K (0.08) | - | + |
| *gra*R | D148Q (0.55) | + | + |
|  | D147E (0.82) | - | + |
| *rpo*B | N474S (0.001) | - | + |
|  | H481Y (0.001) | + | - |
|  | H481N (0.06) | - | + |
|  | S486L (0.001) | - | + |
| *lyt*S | P315R (0.2) | + | - |
|  | A318Q (0.001) | + | - |
|  | A319L(0.001) | + | - |
|  | I320S (0.001) | + | - |
|  | V321M (0.001) | + | - |
| *lyt*R | E31D (0.2) | - | + |
|  | T118N (0.1) | - | + |
|  | G122D (0.9) | - | + |
|  | N125S (0.7) | - | + |
| saeS | L203V (1.0) | - | + |
|  | I340M (0.4) | - | + |
| *pho*R | L144I (0.8) | + | - |
|  | V186I (0.4) | + | - |
|  | V535M (0.03) | + | + |
| *mprF* | T635I (0.3) | - | + |
|  | E709D (0.5) | - | + |
