# Supplementary material for: Genomic insights on heterogeneous resistance to vancomycin and teicoplanin in Methicillin-resistant Staphylococcus aureus: A first report from South India
Source: PLoS One. 2019 Dec 30;14(12):e0227009. doi: 10.1371/journal.pone.0227009 (PMC6936811; doi:10.1371/journal.pone.0227009)
Supplement: S4 Table — (DOCX) [file pone.0227009.s005.docx]

**S4 Table.**

| **Isolate ID** | **Triton X-100 induced autolysis**  **(Absorbance at OD600, %)** | | | **Doubling time (min)** | | |
| --- | --- | --- | --- | --- | --- | --- |
|  | **Mean ± SD** | ***S. aureus* ATCC 29213 (*p-v*alue)** | **MU3**  **(*p -* value)** | **Mean ± SD** | ***S. aureus***  **ATCC 29213**  **(*p-v*alue)** | **MU3**  **(*p -* value)** |
| VB169 | 55.7 ± 25.2 | **0.03** | 0.88 | 33.1 ± 1.7 | 0.14 | 0.35 |
| VB12268 | 55.2 ± 26.5 | **0.04** | 1 | 32.3 ± 1.8 | 0.21 | 0.25 |
| VB26276 | 56.1 ± 24.9 | **0.02** | 0.79 | 33.2 ± 1.7 | 0.13 | 0.36 |
| VB9352 | 62.5 ± 25.4 | **<0.001** | **0.04** | 36.8 ± 1.7 | **0.01** | 0.9 |
| VB23686 | 60.57 ± 25.7 | **0.002** | 0.13 | 36.4 ± 1.6 | **0.02** | 1 |
| VB31683 | 62.8 ± 26.2 | **<0.001** | **0.02** | 37.7 ± 1.7 | **0.01** | 0.8 |
| *S. aureus* ATCC 29213 | 47.8 ± 25.2 | - | - | 27.80 ±1.3 | - | - |
| MU3 | 55.2 ± 21.1 | - | - | 36.36 ±1.7 | - | - |
| MU50 | 65.3 ± 21.8 | - | - | 39.41 ±1.8 | - | - |

*** *p* value <0.05 is considered statistically significant and are in bold face.**
