# Supplementary material for: Genomic insights on heterogeneous resistance to vancomycin and teicoplanin in Methicillin-resistant Staphylococcus aureus: A first report from South India
Source: PLoS One. 2019 Dec 30;14(12):e0227009. doi: 10.1371/journal.pone.0227009 (PMC6936811; doi:10.1371/journal.pone.0227009)
Supplement: S5 Table — (DOCX) [file pone.0227009.s006.docx]

**S5 Table.**

| Isolate ID | Triton X-100 induced autolysis (Absorbance at OD600, %) | | | Doubling time (min) | | |
| --- | --- | --- | --- | --- | --- | --- |
|  | Mean ± SD | *S. aureus* ATCC 29213 (*p-v*alue) | MU3  (*p -* value) | Mean ± SD | *S. aureus*  ATCC 29213  (*p-v*alue) | MU3  (*p -* value) |
| B6832 | 51.9 ± 20.4 | 0.24 | 0.35 | 33.80 ± 1.6 | 0.1 | 0.46 |
| B38761 | 53.9 ± 21.6 | 0.09 | 0.72 | 35.4 ± 1.7 | **0.04** | 0.77 |
| B44746 | 50.8 ± 22.8 | 0.38 | 0.14 | 34.70 ± 1.7 | 0.06 | 0.63 |
| B6907 | 51.5 ± 21.6 | 0.30 | 0.30 | 35.70 ± 1.7 | **0.03** | 0.84 |
| B30101 | 48.2 ± 24.5 | 0.90 | 0.05 | 34.80 ± 1.6 | 0.05 | 0.65 |
| FF1489 | 47.8 ± 22.4 | 0.38 | 0.05 | 33.70 ± 1.8 | 01 | 0.44 |
| FF2016 | 52.4 ± 21.7 | 0.20 | 0.42 | 35.60 ± 1.6 | **0.03** | 0.82 |
| FF507 | 50.9 ± 19.5 | 0.37 | 0.23 | 36.10 ± 1.5 | **0.02** | 0.93 |
| B13333 | 51.5 ± 19.7 | 0.06 | 0.31 | 35.40 ± 1.6 | **0.04** | 0.77 |
| B9882 | 49.6 ± 21.9 | 0.61 | 0.05 | 33.50 ±1.7 | 0.11 | 0.41 |
| BA43964 | 51.5 ± 24.9 | 0.30 | 0.29 | 35.40 ± 1.7 | **0.04** | 0.77 |
| BA46389 | 52.4 ± 21.3 | 0.19 | 0.44 | 34.10 ± 1.7 | 0.08 | 0.51 |
| BA43011 | 50.3 ± 21.5 | 0.47 | 0.18 | 34.90 ± 1.8 | 0.05 | 0.67 |
| CS1919 | 51.7 ± 19.8 | 0.26 | 0.33 | 33.50 ± 1.7 | 0.11 | 0.41 |
| FF1490 | 52.3 ± 22.8 | 0.20 | 0.26 | 34.10 ± 1.7 | 0.08 | 0.51 |
| B20017 | 51.6 ± 24.7 | 0.65 | 0.21 | 36.8 ± 1.7 | **0.01** | 0.09 |
| BA44094 | 49.8 ± 23.1 | 0.57 | 0.22 | 33.40 ± 1.9 | 0.12 | 0.39 |
| B35316 | 49.7 ± 24.6 | 0.57 | 0.13 | 33.60 ± 1.7 | 0.11 | 0.43 |
| B3985 | 49.7 ± 21.3 | 0.58 | 0.13 | 34.20 ± 1.6 | 0.08 | 0.53 |
| BA15100 | 51.6 ± 19.9 | 0.64 | 0.32 | 33.80 ± 1.9 | 0.1 | 0.46 |
| BP3859 | 49.4 ± 21.4 | 0.65 | 0.11 | 33.90 ± 1.6 | 0.09 | 0.48 |
| B7185 | 54.4 ± 21.5 | 0.08 | 0.74 | 36.10 ± 1.7 | **0.02** | 0.93 |
| BA32883 | 52.8 ± 21.8 | 0.16 | 0.51 | 33.20 ± 1.6 | 0.13 | 0.36 |
| BA33052 | 50.7 ± 21.3 | 0.41 | 0.21 | 33.80 ± 1.6 | 0.21 | 0.46 |
| BA16104 | 53.7 ± 21.5 | 0.10 | 0.67 | 34.70 ± 1.5 | 0.06 | 0.63 |
| BA21032 | 54.2 ± 21.1 | 0.08 | 0.77 | 32.40 ± 1.8 | 0.2 | 0.27 |
| BA22553 | 50.6 ± 19.9 | 0.51 | 0.20 | 33.90 ± 1.8 | 0.09 | 0.48 |
| BA13872 | 51.9 ± 22.1 | 0.25 | 0.35 | 33.80 ± 1.9 | 0.1 | 0.46 |
| B14511 | 51.6 ± 21.5 | 0.28 | 0.32 | 34.13 ± 1.6 | 0.08 | 0.52 |
| BA11894 | 50.9 ± 21.4 | 0.38 | 0.23 | 33.50 ± 1.6 | 0.11 | 0.41 |
| BA31796 | 49.5 ± 20.9 | 0.61 | 0.12 | 33.90 ± 1.8 | 0.09 | 0.48 |
| B4283 | 49.4 ± 22.9 | 0.72 | 0.09 | 35.40 ± 1.8 | **0.04** | 0.77 |
| BA6415 | 51.6 ± 20.9 | 0.28 | 0.32 | 34.90 ± 1.7 | 0.05 | 0.67 |
| BA3792 | 50.8 ± 21.4 | 0.39 | 0.22 | 35.70 ± 1.6 | **0.03** | 0.84 |
| BP3820 | 54.9 ± 22.5 | 0.05 | 0.93 | 34.50 ± 1.9 | 0.06 | 0.59 |
| BP834 | 49.5 ± 21.6 | 0.59 | 0.12 | 34.80± 1.5 | 0.05 | 0.65 |
| BA5031 | 51.4 ± 21.3 | 0.30 | 0.29 | 35.30 ± 1.6 | **0.04** | 0.75 |
| BA5309 | 49.7 ± 20.5 | 0.16 | 0.13 | 35.20 ± 1.5 | **0.04** | 0.73 |
| BA10785 | 50.1 ± 21.4 | 0.50 | 0.16 | 34.63 ± 1.7 | 0.06 | 0.61 |
| BA14915 | 53.8 ± 21.5 | 0.14 | 0.54 | 35.31 ± 1.9 | **0.04** | 0.75 |
| BP5187 | 54.2 ± 22.8 | 0.08 | 0.78 | 34.90 ± 1.9 | 0.05 | 0.67 |
| BA1801 | 53.6 ± 21.1 | 0.11 | 0.65 | 35.60 ± 1.8 | **0.03** | 0.82 |
| BP13568 | 53.5 ± 21.5 | 0.11 | 0.63 | 35.70 ± 1.9 | **0.03** | 0.84 |
| BA21900 | 51.4 ± 21.7 | 0.30 | 0.29 | 35.10 ± 1.8 | 0.05 | 0.71 |
| BA24325 | 52.5 ± 20.7 | 0.18 | 0.45 | 34.80 ± 1.9 | 0.05 | 0.65 |
| BA12804 | 51.3 ± 21.6 | 0.36 | 0.24 | 34.30 ± 1.7 | 0.07 | 0.55 |
| BA24023 | 51.5 ± 21.3 | 0.30 | 0.3 | 33.10 ± 1.5 | 0.14 | 0.35 |
| BA33868 | 49.9 ± 20.5 | 0.54 | 0.14 | 33.50 ± 1.8 | 0.1 | 0.41 |
| BA103 | 52.3 ± 20.7 | 0.21 | 0.41 | 33.60 ± 1.6 | 0.11 | 0.43 |
| BA14468 | 51.6 ± 20.5 | 0.28 | 0.32 | 33.70 ± 1.8 | 0.1 | 0.44 |
| BP7336 | 53.1 ± 21.5 | 1.40 | 0.56 | 36.10 ± 1.5 | **0.02** | 0.93 |
| BA5275 | 50.4 ± 21.8 | 0.53 | 0.15 | 33.20 ± 1.8 | 0.13 | 0.36 |
| BA20860 | 53.7 ± 21.3 | 0.10 | 0.67 | 35.80 ± 1.6 | **0.03** | 0.86 |
| B9939 | 50.3 ± 21.4 | 0.49 | 0.16 | 34.80 ± 1.7 | 0.05 | 0.65 |
| B16578 | 52.4 ±19.6 | 0.19 | 0.38 | 33.7 ± 1.6 | 0.20 | 0.46 |
| B9190 | 53.1 ±21.5 | 0.10 | 1.40 | 34.8 ± 1.8 | 0.09 | 0.67 |
| BA35739 | 51.7 ± 19.3 | 0.40 | 0.30 | 33.2 ±1.9 | 0.13 | 0.36 |
| BA25679 | 53.6 ± 1.2 | 0.14 | 0.11 | 34.7 ± 1.8 | 0.05 | 0.67 |
| *S. aureus* ATCC 29213 | 47.8 ± 25.2 | - | - | 27.80 ±1.3 | - | - |
| MU3 | 55.2 ± 21.1 | - | - | 36.36 ±1.7 | - | - |
| MU50 | 65.3 ± 21.8 | - | - | 39.41 ±1.8 | - | - |

*** *p* value <0.05 is considered statistically significant and are in bold face.**
