# Supplementary material for: Genomic insights on heterogeneous resistance to vancomycin and teicoplanin in Methicillin-resistant Staphylococcus aureus: A first report from South India
Source: PLoS One. 2019 Dec 30;14(12):e0227009. doi: 10.1371/journal.pone.0227009 (PMC6936811; doi:10.1371/journal.pone.0227009)
Supplement: S6 Table — (DOCX) [file pone.0227009.s007.docx]

**S6 Table.**

| **Isolate ID** | **Amino acid substitutions** | |
| --- | --- | --- |
|  | ***pbp*2** | ***pbp*4** |
| VB9352 | P285A  T439V | D98E  T253P |
| VB23086 | T439V | D98E |
| VB31683 | A420V  A557T  T691A | D98E  T253P |
| VB26276 | T439V | D98E |
| VB12268 | - | - |
| VB169 | - | - |
|  |  |  |
| VB4283 | N81S  P285A | **-** |
| VB3985 | P825A | E398A |
| VB1919 | T439V  T691A | E398A |
| VB35316 | - | T25A  Q283A |
| VB43011 | P825A | S395C |
| VB43964 | P825A  T439V  T489E  T691A | T25A  E398A |
| VB1490 | H121R | S395C  A409T |
| VB9882 | P825A  T439V  T489E  T691A | T25A  D98E  E398A |
| VB20017 | P825A  T439V  T489E  T691A | T25A  D98E  E398A |
| VB44094 | P285A  T439V T489E  T691A | T25A  D98E  E398A |
| VB44746 | P825A | T25A  E398A |
| VB9939 | P285A  T439V | D98E  T253P |
| VB16578 | P285A  T439V | D98E  T253P |
| VB46389 | P285A | **-** |
| VB7336 | P285A | D98E  T253P |
| VB13872 | P285A | D98E  T253P |
| VB14468 | P285A | D98E  T253P |
| BA14915 | P285A | D98E  T253P |
| VB103 | P285A | D98E  T253P |
| VB9190 | R262C | A409T |
| VB14511 | R262C | **-** |
| VB25679 | P285A  T439V  T489E  T691A | T25A  D98E  E398A |
| VB7185 | A172T | S395C  A409T |
