# Supplementary material for: Genomic insights on heterogeneous resistance to vancomycin and teicoplanin in Methicillin-resistant Staphylococcus aureus: A first report from South India
Source: PLoS One. 2019 Dec 30;14(12):e0227009. doi: 10.1371/journal.pone.0227009 (PMC6936811; doi:10.1371/journal.pone.0227009)
Supplement: S7 Table — (DOCX) [file pone.0227009.s008.docx]

**S7 Table.**

|  | **Isolate ID** | **Sequence Type** | **Accession no.** |
| --- | --- | --- | --- |
| Reduced teicoplanin susceptible MRSA | VB9352 | ST772 | LXWR00000000 |
|  | VB23686 | ST772 | MANS00000000 |
|  | VB26276 | ST772 | LWMF00000000 |
|  | VB12268 | ST672 | LXWS00000000 |
|  | VBV169 | ST672 | LWMG00000000 |
|  | VB31683 | ST22 | MANT00000000 |
| hVISA | VB14511 | ST239 | RJSA00000000 |
|  | VB25679 | ST22 | RJSC00000000 |
|  | VB7185 | ST368 | RJSB00000000 |
|  | VB9190 | ST239 | RJSD00000000 |
|  | VB9939 | ST772 | MLQK00000000 |
|  | VB16578 | ST772 | MLQD00000000 |
|  | VB46389 | ST772 | MLQG00000000 |
|  | VB7336 | ST772 | RHIH00000000 |
|  | VB14915 | ST772 | RHIS00000000 |
|  | VB103 | ST772 | RHIC00000000 |
|  | CS1919 | ST22 | RXZG00000000 |
|  | VB44094 | ST22 | MLQH00000000 |
|  | VB9882 | ST2371 | MLQI00000000 |
|  | VB20017 | ST2371 | MLQE00000000 |
|  | VB1490 | ST239 | MLQB00000000 |
|  | VB43011 | ST1 | MLQJ00000000 |
|  | VB44746 | ST1290 | MLQF00000000 |
|  | VB4283 | ST1482 | [NBSI00000000](https://www.ncbi.nlm.nih.gov/nuccore/NBSI00000000) |
|  | BA43964 | ST580 | [MLQA00000000](https://www.ncbi.nlm.nih.gov/nuccore/MLQA00000000) |
|  | VB3985 | ST6 | [NBSJ00000000](https://www.ncbi.nlm.nih.gov/nuccore/NBSJ00000000) |
|  | VB35316 | ST72 | MLQC00000000 |
|  | VB13872 | ST772 | RHIO00000000 |
|  | VB14468 | ST772 | RHIQ00000000 |
